# Realization of tristability in a multiplicatively coupled dual-loop genetic network

**DOI:** 10.1038/srep28096

**Published:** 2016-07-05

**Authors:** Bo Huang, Yun Xia, Feng Liu, Wei Wang

**Affiliations:** 1 National Laboratory of Solid State Microstructures, Department of Physics, and Collaborative Innovation Center of Advanced Microstructures, Nanjing University, Nanjing 210093, China

## Abstract

Multistability is a crucial recurring theme in cell signaling. Multistability is attributed to the presence of positive feedback loops, but the general condition and essential mechanism for realizing multistability remain unclear. Here, we build a generic circuit model comprising two transcription factors and a microRNA, representing a kind of core architecture in gene regulatory networks. The circuit can be decomposed into two positive feedback loops (PFLs) or one PFL and one negative feedback loop (NFL), which are multiplicatively coupled. Bifurcation analyses of the model reveal that the circuit can achieve tristability through four kinds of bifurcation scenarios when parameter values are varied in a wide range. We formulate the general requirement for tristability in terms of logarithmic gain of the circuit. The parameter ranges for tristability and possible transition routes among steady states are determined by the combination of gain features of individual feedback loops. Coupling two PFLs with bistability or one NFL with a bistable PFL is most likely to generate tristability, but the underlying mechanisms are largely different. We also interpret published results and make testable predictions. This work sheds new light on interlinking feedback loops to realize tristability. The proposed theoretical framework can be of wide applicability.

Bistability and multistability are common dynamic features of a wide variety of natural and synthetic systems^1^. A multistable system can switch between alternative stable states in response to external inputs. Multistability plays a key role in diverse biological processes such as cell differentiation^2^,^3^ and epithelial to mesenchymal transition (EMT)^4^,^5^,^6^,^7^, allowing for cell-fate determination among multiple phenotypes. Alternative differentiated states in development may correspond to multiple stable steady states of regulatory networks, while the hybrid phenotype in EMT can be interpreted as an intermediate state of a tristable circuit.

Positive feedback is necessary for bistability and multistability^1^,^8^. A positive feedback loop (PFL) with ultrasensitivity is able to exhibit bistability^9^; a single PFL seems insufficient to admit multistability^10^, but coupling a PFL with additional feedback loops is possible to realize multistability^11^,^12^,^13^,^14^. Indeed, tristability or tetrastability can be induced in interlinked PFLs, which are at the core of signaling pathways underlying the EMT^4^,^5^,^6^,^7^,^15^. Notably, these systems involve transcriptional coregulation of a microRNA (miRNA) and its targets, representing a recurrent motif in gene regulatory networks. Typical interlinked dual-PFLs include a mutual inhibitory loop (MIL) combined with a self-activation loop^4^ (Fig. 1A), two MILs sharing one component^7^ (Fig. 1B), and one component in an MIL inhibiting the other through a feed-forward loop^4^,^5^,^6^,^7^ (Fig. 1C). The first two structures have been investigated in detail^15^,^16^, whereas the last one was less probed. It is worth exploring since similar architectures are widely involved in cell signaling^17^,^18^,^19^, such as that responsible for the differentiation of human embryonic stem cells^18^ (Fig. 1D). Moreover, whether coupling a PFL with a negative feedback loop (NFL) can induce tristability is an open issue. Rather than exploring the underlying mechanism for realizing tristability in interlinked dual loops on a case-by-case basis, it is necessary to establish a general principle. It is essential to reveal how the features of individual feedback loops and their coupling manner affect the tristability.

Based on the circuits in Fig. 1C,D, here we construct a circuit model comprising two transcription factors (TFs) and one miRNA. Mathematical equations characterizing the temporal evolution of circuit components take canonical forms. First, we explore whether the circuits of individual or coupled feedback loops can exhibit tristability over a wide range of parameter values by bifurcation analysis. Second, we introduce the measure of logarithmic gain and formulate the necessary condition for tristability in terms of the gain. Third, we investigate six representative systems (i.e., the circuits with different parameter values) and illustrate the requirements for tristability by presenting both analytical and numerical results. Finally, we discuss our methods, main results, and their relevance to cell signaling. This work reveals the essential mechanism for realizing tristability in multiplicatively coupled feedback loops.

## Model and Method

**Figure 1:**
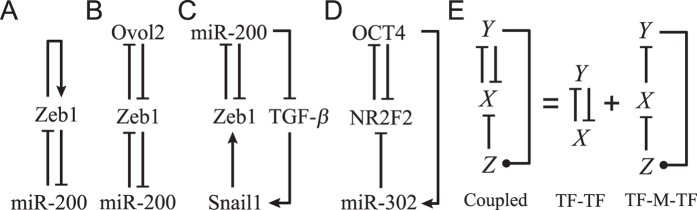
Interlinked feedback loops. (**A–D**) Examples of miRNA-containing circuits where two loops share one (**A**,**B**) or two (**C**,**D**) components. (**E**) The model. The system of coupled feedback loops can be decomposed into two loops. The arrow- and bar-headed lines denote activation and inhibition, respectively, while circle-headed lines denote either activation or inhibition.

The circuit model comprises three nodes termed *X*, *Y* and *Z* (Fig. 1E). *X* and *Y* are two TFs, while *Z* is a miRNA (M). Without loss of generality, we assume that two TFs mutually repress each other’s transcription, forming a TF-TF PFL. *Y* regulates the expression of *Z* and *Z* represses the production of *X*, constituting a TF-M-TF loop. This loop is either a PFL or an NFL, depending on whether *Y* activates or inhibits the transcription of *Z*. Furthermore, the sign of the TF-M-TF loop determines whether the coupled system is a PP (PFL-PFL) or PN (PFL-NFL) circuit (note that a dual-NFL circuit cannot admit multistability). The time evolution of the system is governed by the following ordinary differential equations:


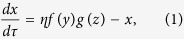



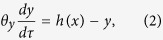



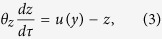


where 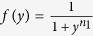, 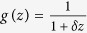, 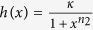, and 
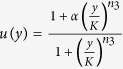
. All variables and parameters are normalized to be dimensionless (see *SI Text* 1 for original equations and the derivation).

The variables *x*, *y* and *z* denote the concentrations of *X*, *Y* and *Z*, respectively. The term *ηf*(*y*)*g*(*z*) describes the production rate of *X*; *f*(*y*) denotes the repressive regulation by *Y* at the transcriptional level, while *g*(*z*) characterizes the inhibition by *Z* at the translational level. The product of *f*(*y*) and *g*(*z*) characterizes the coregulation of *X* by *Y* and *Z*, representing a multiplicative coupling of two loops. *η* is the maximum production rate, taken as a control parameter here, and *δ* in *g*(*z*) reflects the strength of inhibition by *Z*. Similarly, *h*(*x*) describes the production rate of *Y*; *κ* is the maximum production rate. *u*(*y*) describes the regulation of *Z* transcription by *Y*; *K* relates to the dissociation constant for *Y* binding to the promoter of *Z*, while *α* > 1 and *α* < 1 represent the degree to which transcription is enhanced or weakened, respectively, by *Y* bound to the promoter. All the negative terms describe the process of degradation. *n*_1_, *n*_2_ and *n*_3_ are Hill coefficients, *τ* is the time in units of the reciprocal of the degradation rate of *X*, and 1/*θ*_*y*_ and 1/*θ*_*z*_ are the degradation rates of *Y* and *Z* normalized to that of *X*, respectively.

If we eliminate the indirect regulation of *X* by *Y* via *Z*, we get the equations for the TF-TF loop directly from the above equations:


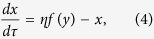


and Eq. 2. If we ignore the direct regulation of *X* by *Y*, we get the equations for the TF-M-TF loop:


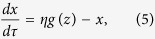


and Eqs 2 and 3. The parameter values are not fixed; instead, we explore the properties of these systems over a wide parameter range. Numerical simulations are performed using MATLAB 2008b. Bifurcation diagrams are plotted using Oscill8.

## Results

### Analysis of individual and coupled feedback loops

We first demonstrate that only the circuits of coupled feedback loops can admit tristability via bifurcation analysis. 50000 parameter sets are sampled for the PP (*α* > 1) and PN (*α* < 1) circuits, respectively, using the Latin hypercube sampling method (MATLAB built-in function *lhsdesign*). The ranges for parameter sampling are listed in Table S1. For each parameter set, we plot the steady-state value of *x* versus *η*.

**Figure 2:**
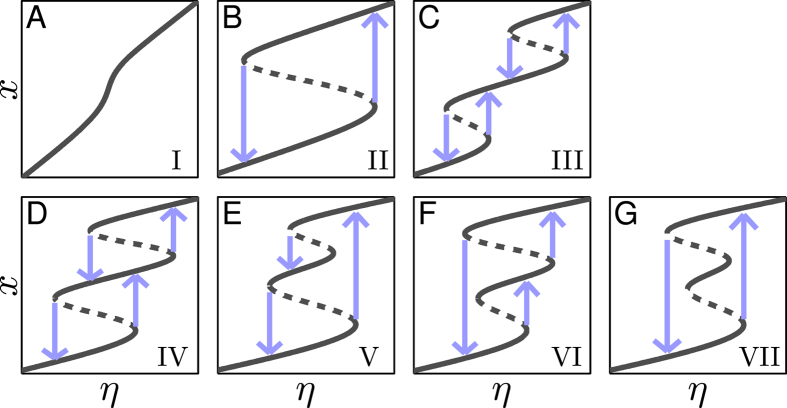
Schematic description of seven types of bifurcation diagrams for both the PP and PN circuits. Types I and II and type I are allowable for individual PFLs and NFLs, respectively. The solid and dashed lines denote stable and unstable steady states, respectively. The blue lines indicate possible transitions between steady states via SN bifurcation. Both axes are in log scale.

For individual PFLs and PP circuits, all the bifurcation diagrams are categorized into seven types (Fig. 2). For the TF-TF or TF-M-TF PFL, only type I and type II are allowable, involving monostability and bistability (Fig. 2A,B). Thus, individual PFLs in our work cannot exhibit tristability. For convenience, we denote the PFL with the bifurcation diagram of type I or II by P_I_ and P_II_, respectively. Therefore, PP circuits are grouped into four categories: P_I_P_I_, P_I_P_II_, P_II_P_I_ and P_II_P_II_ (the first item denotes the type of the TF-TF loop and the second denotes that of the TF-M-TF loop). Notably, PP circuits can admit five more types of bifurcation diagram besides types I and II: type III involves two separate bistability regions (Fig. 2C), while types IV–VII each have one tristability region, which is adjoined to two bistability regions, with different bifurcation points (Fig. 2D–G). As seen in Table S2, P_II_P_II_ is the most probable one to achieve tristability among PP circuits (with a probability of 0.37).

For individual NFLs and PN circuits, there appear more types of bifurcation diagram owing to the presence of Hopf bifurcation, via which some stable steady states become unstable. Fortunately, these unstable steady states can always be restabilized by adjusting parameters *θ*_*y*_ and *θ*_*z*_ to eliminate Hopf bifurcation (see *SI Text* 2). Consequently, all the bifurcation diagrams can be converted to the seven types illustrated in Fig. 2. This greatly simplifies the search for PN circuits with tristability. In such a setting, individual NFLs admit only the bifurcation diagram of type I, and PN circuits are divided into P_I_N and P_II_N (‘N’ denotes the NFL). Only type I is allowed for P_I_N circuits, whereas types I–VII are observable for P_II_N ones. In the following, we focus on how to realize tristability in the P_II_P_II_ and P_II_N circuits.

### Necessary condition for tristability in terms of gain

To quantify the essential condition for tristability, we tried multiple measures and found that logarithmic gain of circuits is an ideal candidate. Generally, the gain of a system not only reflects the effect of feedback like amplification or attenuation, but also tightly associates with the elements of the system’s Jacobian matrix, which govern the stability of steady states^20^,^21^. For individual loops, the logarithmic gain (denoted by *G* and called gain for short thereafter) is defined as the derivative of a transfer function (*T*) with respect to *x* in log-log coordinates. The transfer function is the composition of functions, each describing one of regulations in a feedback loop (see *SI Text* 3). Thus, the gain depends on all regulatory interactions in one loop. For the TF-TF loop, the transfer function and the gain are separately *T*_1_(*x*) = (*ηf*◦*h*)(*x*) and 
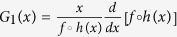
. For the TF-M-TF loop, the transfer function and the gain are *T*_2_(*x*) = (*ηg*◦*u*◦*h*)(*x*) and 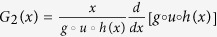, respectively.

**Figure 3:**
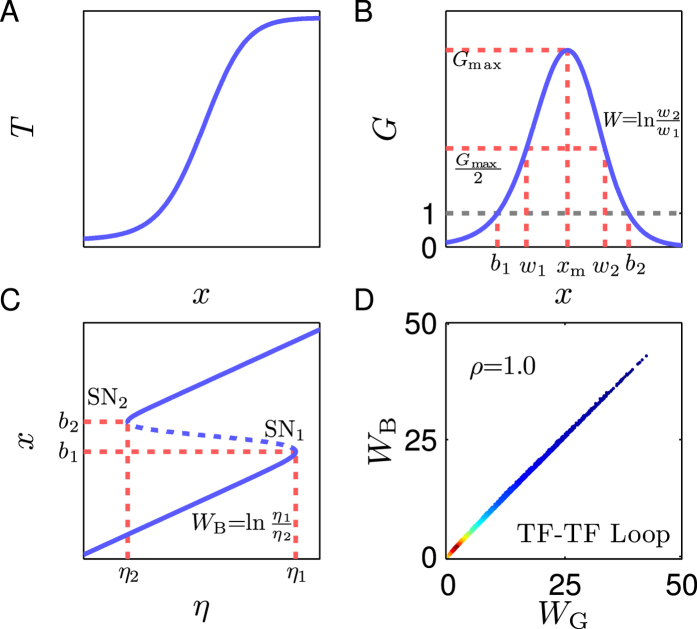
Characteristics of individual PFLs in terms of gain. (**A**) Transfer function *T* is characterized by a sigmoid function. (**B**) Logarithmic gain *G*. If *G*_max_ > 1, *G* equals 1 at *b*_1_ and *b*_2_. (**C**) Bifurcation diagram. Two SN bifurcations (marked by SN_1_ and SN_2_) appear at *η*_1_ and *η*_2_. (**D**) Scatter diagram of *W*_B_ versus *W*_G_. Each dot represents one TF-TF loop. From blue to red, more dots are distributed locally. All axes except the *G*-axis are in log scale in (**A–C**).

A typical transfer function *T*(*x*) for the TF-TF or TF-M-TF PFL is a sigmoid function characterizing the involved cooperativity and saturation^22^,^23^ (Fig. 3A), while that for the TF-M-TF NFL is a reverse sigmoid function (Fig. S1A). The corresponding *G*(*x*) curve for the PFL is bell shaped with one positive maximum (*G*_max_) at *x*_m_ (Fig. 3B), while that for the NFL is inverted bell shaped with one negative minimum (*G*_min_) at *x*_m_ (Fig. S1B). Three features of the gain are used to represent individual loops, i.e., *G*_max_ (*G*_min_), *x*_m_, and the full width *W* at half extremum. Since the (reverse) sigmoid function has the steepest slope at *x*_m_, *x*_m_ is called activation threshold, especially when *T*(*x*) is close to a threshold-like function. *W* is defined as ln(*w*_2_/*w*_1_) with *w*_1_ and *w*_2_ denoting the *x* values at half extremum points. Notably, feedback loops with a bell-shaped *G*(*x*) seem to have a ‘bandpass’ property: only within a limited concentration range is the gain significantly nonzero; otherwise, it decays to zero rapidly. This ‘bandpass’ property has a critical role in shaping the feature of the system of coupled feedback loops as seen later.

Using the standard bifurcation analysis, we obtain a necessary condition for saddle-node (SN) bifurcation (see *SI Text* 3): if a dynamic system undergoes an SN bifurcation at a steady state denoted by *x*, the gain at *x* must be 1, i.e., *x* is a solution to the equation *G*(*x*) = 1. For the system to exhibit bistability, it must undergo two SN bifurcations, i.e., there should be two distinct real solutions to *G*(*x*) = 1. Similarly, the necessary condition for tristability is that there should be four distinct real solutions to *G*(*x*) = 1. For general multistability, *G*(*x*) = 1 should have at least four different real solutions. Of note, here we omit other types of bifurcation like pitchfork bifurcation because the appearance of bistability and tristability in the above systems all originates from SN bifurcation (Fig. 2), which is typical of cell-fate decision^5^,^15^,^24^.

For any PFL with *G*_max_ > 1, there are at most two solutions to *G*(*x*) = 1 due to the bell-shaped *G*(*x*) curve; thus, the PFL can undergo at most two SN bifurcations and display bistability in some range of *η*, never admitting tristability (Fig. 3C). The width of the bistability region is defined as the logarithmic difference between its upper and lower thresholds, *W*_B_ = ln(*η*_1_/*η*_2_). Notably, *W*_B_ can be approximately expressed as follows (using an isosceles triangle to approximate the gain curve; see *SI Text* 4.1 for details):


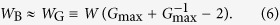


To verify this, we separately sample 6200 and 5260 parameter sets for the TF-TF and TF-M-TF PFLs with bistability and calculate *W*_B_, *G*_max_ and *W* for each case. The points in the (*W*_G_, *W*_B_) plane indeed scatter along the diagonal, with the Pearson’s correlation coefficient being 1.0 (Figs 3D and S2).

### Difference between activation thresholds affects the presence of tristability in P_II_P_II_ circuits

If we apply the definitions of transfer function and gain to the system of coupled feedback loops, we have *T*_C_(*x*) = [*η*(*f*◦*h*)(*g*◦*u*◦*h*)](*x*) and *G*_C_(*x*) = *G*_1_(*x*) + *G*_2_(*x*) (see *SI Text* 3), i.e., the gain of the coupled system is the sum of the gains of individual feedback loops. This additivity results from the product of functions when describing the regulation of *X* by *Y* and *Z*, i.e., the multiplicative coupling. Given the *G*_C_(*x*) curve is a superposition of *G*_1_(*x*) and *G*_2_(*x*) curves, the difference between *x*_1m_ and *x*_2m_ markedly affects the shape of *G*_C_(*x*) and presence of tristability. Similar to the definitions of *W* and *W*_B_, a logarithmic difference is defined, i.e., *R* = ln(*x*_2m_/*x*_1m_). To focus on the influence of *R* on tristability, we merely vary *R* while keeping other factors unchanged. Without loss of generality, we develop a numerical method in which three parameters *n*_3_, *α*, and *K* are adjusted simultaneously such that only *x*_2m_ is changed (see *SI Text* 5). In the following, we present only the results for the case of 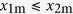, i.e., 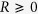 (we also investigated the case of *R* < 0, and similar conclusions are drawn).

**Figure 4:**
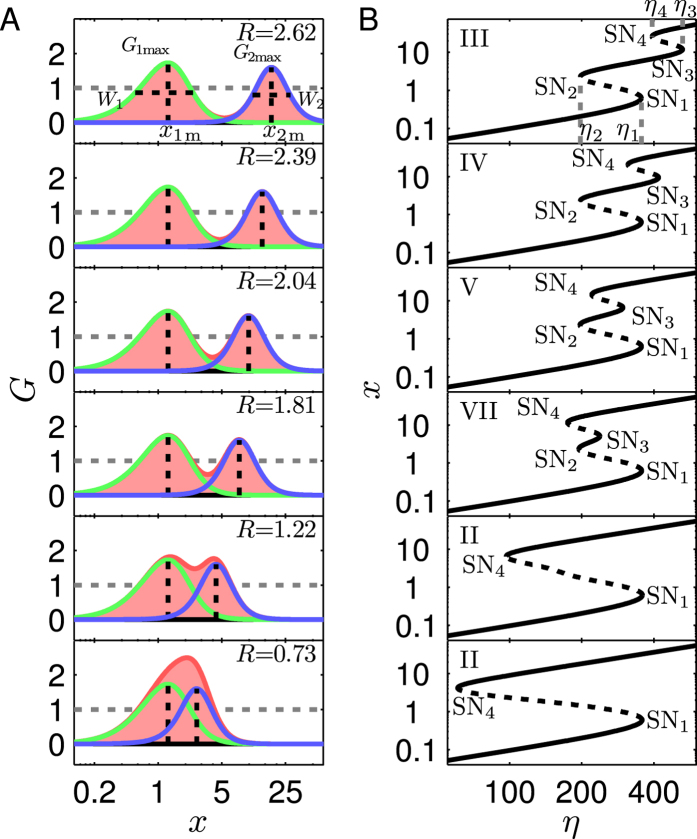
Effects of *R* on *G*_*C*_ and bifurcation diagrams. *x*_1m_ is fixed at 1.28, while *x*_2m_ varies with *R* (System 1). (**A**) The curves of *G*_1_ (green), *G*_2_ (blue) and *G*_C_ (red); (**B**) the corresponding bifurcation diagrams, where the solid and dashed curves denote stable and unstable steady states, respectively. The four bifurcation points and the corresponding *η* values are marked. The *x*-axis and *η*-axis are in log scale.

A representative system is chosen with *G*_1max_ = 1.73, *x*_1m_ = 1.28, *W*_1_ = 1.46, *G*_2max_ = 1.60, and *W*_2_ = 0.96 (for parameter values see Table S3, System 1). When *R* is relatively large (e.g., *R* = 2.62), the *G*_C_(*x*) curve has two local maxima and one local minimum, intersecting with the line *G* = 1 four times (Fig. 4A). Accordingly, four SN bifurcation points appear at *η*_*i*_(i = 1 ~ 4) in the bifurcation diagram (Fig. 4B). However, no tristability region arises, but two separate bistability regions appear (i.e., the bifurcation diagram is of type III). Actually, the widths of two bistability regions, *W*_BC1_ = ln(*η*_1_/*η*_2_) and *W*_BC2_ = ln(*η*_3_/*η*_4_), approximately equal those for the TF-TF and TF-M-TF loops (i.e., *W*_B1_ and *W*_B2_), respectively (*SI Text* 4.2). Based on Eq. 6, we further have


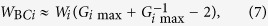


with *i* = 1, 2. On the other hand, the width of the intermediate branch of stable steady states (bounded by SN_2_ and SN_3_), *W*_I_ = ln(*η*_3_/*η*_2_), obeys (*SI Text* 4.3):


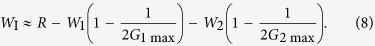


If *R* is so large that *W*_I_ > *W*_BC1_ + *W*_BC2_, two bistability regions remain separate. In this case, the coupled system acts as a bistable switch within two distinct ranges of *η*, determined by the features of individual loops.

For a smaller *R* (e.g., *R* = 2.39, 2.04, or 1.81), the *G*_C_(*x*) curve still has two local maxima and one local minimum and intersects with *G* = 1 four times. While Eqs 7, 8 still hold true, the reduction in *R* leads to *W*_I_ < *W*_BC1_ + *W*_BC2_. Consequently, two bistability regions overlap, resulting in a tristability region. Accordingly, the bifurcation diagrams are separately of type IV, V, and VII. If *R* further drops (e.g., *R* = 1.22), however, the local minimum of *G*_C_(*x*) between two local maxima is elevated above 1, thus leaving only two intersections with *G* = 1. Consequently, there exist only two SN bifurcations and tristability is no longer possible. For *R* = 0.73, there is even no local minimum, and the *G*_C_(*x*) curve is single-bell shaped. The coupled system acts as a bistable switch in a wider range of *η*. We conclude that only when *R* is moderate, the bistability properties of two PFLs can be combined to generate tristability.

**Figure 5:**
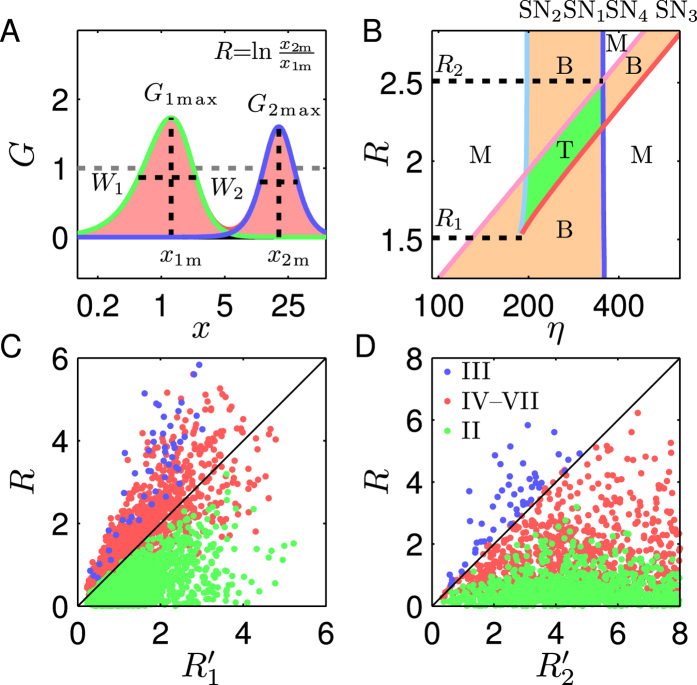
Dependence of SN bifurcation points on *R* and *η*. (**A**) The curves of *G*_1_ (green), *G*_2_ (blue) and *G*_C_ (red). (**B**) Phase diagram of *R* versus *η*. The solid curves denote the loci of bifurcation points: SN_1_ (blue), SN_2_ (cyan), SN_3_ (red) and SN_4_ (magenta). The white, orange, and green domains correspond to monostability, bistability, and tristability, respectively. The *x*-axis and *η*-axis are in log scale. Scatter diagrams of *R* versus 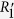 (**C**) or 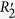 (**D**). Each blue, red or green dot represents one coupled system with the bifurcation diagram of type III, IV–VII and II, respectively.

To quantify the range of *R* for tristability, we plot a phase diagram of *R* versus *η*, where four loci of SN bifurcation points are traced out, dividing the (*η*, *R*) plane into several domains, corresponding to three dynamic behaviors: monostability, bistability and tristability (Fig. 5B). Tristability exists in a limited range of *R*: its lower limit *R*_1_ is 1.51, determined by the intersection of the loci of SN_2_ and SN_3_, while its upper limit *R*_2_ is 2.51, determined by that of SN_1_ and SN_4_. In contrast, only two bistability regions may appear for *R* > *R*_2_, or one bistability region for 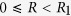 (besides two monostability regions). Thus, for the dual-PFL circuit to exhibit tristability, *R* should be kept within a proper range.

Furthermore, we derive the estimates of *R*_1_ and *R*_2_, 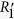 and 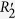, given *G*_1max_ > 1 and *G*_2max_ > 1 (*SI Text* 4.4):


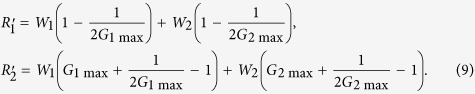


The width of *R*’s range for tristability thus obeys 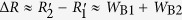. Therefore, the wider the bistability regions for two PFLs, the broader the range of *R* for tristability. To validate Eq. 9, we sample 3200 parameter sets for the coupled system with *G*_1max_ > 1, *G*_2max_ > 1, and 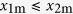, and calculate 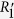, 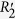, and *R* for each case. For the systems with the bifurcation diagram of type II, nearly all points scatter in the region of 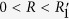 (Fig. 5C), while for those of type III, the points spread in the region of 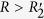 (Fig. 5D); for those of type IV–VII, the points are mainly distributed in the region of 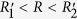. Thus, the range of *R* for tristability is indeed approximately determined by the features of individual PFLs. This has important implications for inducing tristability in coupled feedback loops.

### *W*_B_ of individual PFLs significantly affects the bifurcation type of the coupled system

It is worthy to note that *W*_B1_ and *W*_B2_ affect the bifurcation type of the coupled system. For example, if *W*_B1_ is decreased by setting *G*_1max_ and *W*_1_ to 1.49 and 1.44 and *W*_B2_ is increased by setting *G*_2max_ and *W*_2_ to 2.00 and 0.99 (see Table S3, System 2), then *W*_B1_ is smaller than *W*_B2_, leading to *W*_BC1_ < *W*_BC2_. Consequently, the bifurcation diagram of type V, which requires *W*_BC1_ > *W*_BC2_, no longer occurs given 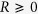 (Fig. S3). Instead, type VI can be observed for some values of *R* (e.g. *R* = 2.14). That is, type V and type VI are mutually exclusive, and which type occurs depends on *W*_B1_ and *W*_B2_. Together, the width of the bistability region for individual PFLs greatly influences the type of bifurcation diagram that the coupled system can admit.

### *W*, *R* and *G*_min_ affect the tristability of P_II_N circuits

Here, we probe how to produce tristability in P_II_N circuits with *G*_1max_ + *G*_2min_ < 1. Intuitively, an NFL may go against bistability since its negative gain leads to *G*_C_ < *G*_1_. For a representative P_II_N circuit with *G*_1max_ = 2.59, *x*_1m_ = 1.81, *W*_1_ = 1.62, *G*_2min_ = −3.70, *x*_1m_ = 1.77 and *W*_2_ = 1.37 (see Table S3, System 3), *G*_C_ is less than 1 (Fig. S4A), and bistability is eliminated (Fig. S4C). In fact, this occurs when *W*_1_ and *W*_2_ are comparable or *W*_2_ is much larger than *W*_1_. If *W*_2_ is much less than *W*_1_, e.g., *W*_2_ = 0.39 (for parameter values see Table S3, System 4), the modulation of *G*_1_ by *G*_2_ can be much localized (Fig. S4B). Only the part around *x*_2m_ is quenched by *G*_2_, and the others nearly remain unchanged. Consequently, *G*_C_(*x*) can have two local maxima (>1) and one local minimum (<1), and a tristability region arises in the bifurcation diagram (Fig. S4D). Thus, the *G*(*x*) curve for the NFL should be much narrower than that for the PFL so as to realize tristability.

**Figure 6:**
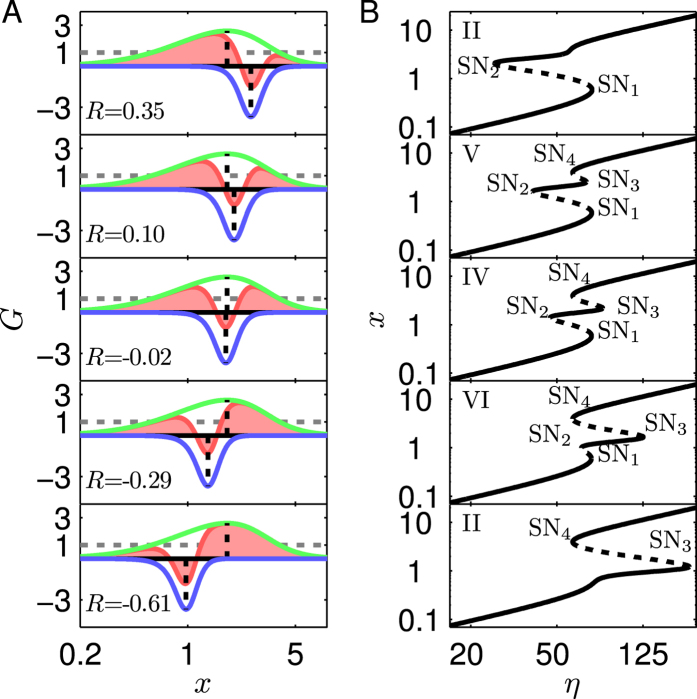
Effects of *R* on *G*_*C*_ and bifurcation diagrams of P_II_N (System 4). (**A**) The curves of *G*_1_ (green), *G*_2_ (blue) and *G*_C_ (red) for different values of *R*; (**B**) the corresponding bifurcation diagrams, where the solid and dashed lines denote stable and unstable steady states, respectively. The *x*-axis and *η*-axis are in log scale.

**Figure 7:**
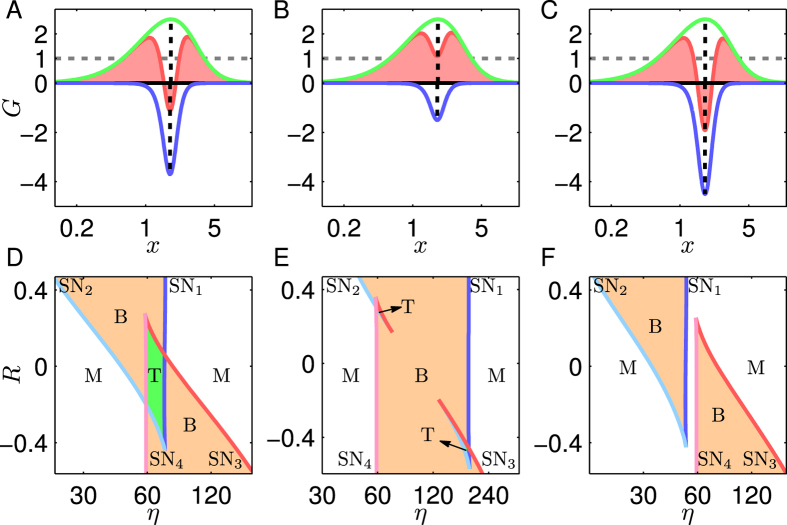
Dependence of bifurcation points on *R* and *η* for P_II_N. (**A–C**) The curves of *G*_1_ (green), *G*_2_ (blue) and *G*_C_ (red); (**D–F**) the (*R*, *η*) phase diagrams. The solid curves denote the loci of SN bifurcation points: SN_1_ (blue), SN_2_ (cyan), SN_3_ (red) and SN_4_ (magenta). The white, orange, and green domains correspond to monostability, bistability, and tristability, respectively. (**A,D**), (**B,E**) and (**C,F**) correspond to Systems 4, 5, 6, respectively. The *x*-axis and *η*-axis are in log scale.

Take System 4 as an example to explore how *R* affects the tristability. With *R* = 0.10, −0.02, or −0.29, *G*_1_ is modulated locally in a similar way (Fig. 6A). As expected, a tristability region exists in the corresponding bifurcation diagrams (Fig. 6B). However, if *R* rises to 0.35 or drops to −0.61, one local maximum of *G*_C_ becomes less than 1, and there are only two solutions to *G*_C_(*x*) = 1. Thus, no tristability can be realized. An overview of *R*’s influence is presented in the (*R*, *η*) phase diagram (Fig. 7A,D). The tristability arises within a limited range of *R*. Its upper limit is 0.28, determined by the intersection of the loci of SN_3_ and SN_4_; the lower limit is −0.45, determined by that of SN_1_ and SN_2_. For *R* > 0.28 or *R* < −0.45, only one bistability and two monostability regions are observable. Therefore, a tristability region appears when *R* approaches 0. This is in contrast to the condition for tristability in P_II_P_II_ circuits; instead, two PFLs with a small |*R*| seem to merge into one PFL, unable to admit tristability. Collectively, for P_II_N with *G*_1max_ + *G*_2min_ < 1 to exhibit tristability, *W*_2_ needs to be much less than *W*_1_, and |*R*| should be sufficiently small.

If the negative feedback is weakened by increasing *G*_2min_ from −3.70 to −1.5 such that *G*_1max_ + *G*_2min_ > 1 (Fig. 7B; for parameter values see Table S3, System 5), the tristability can appear when |*R*| is moderate rather than close to 0 (Figs 7E and S5). However, the tristability regions are rather small, and it is difficult for such systems to exhibit robust tristability. Actually, if *G*_2min_ further rises, there is no tristability region any more. Conversely, if the negative feedback is enhanced by decreasing *G*_2min_ from −3.70 to −4.50 (Fig. 7C; for parameter values see Table S3, System 6), the bifurcation diagram is always of type II or III (Fig. S6), and no tristability appears at any *R* or *η* (Fig. 7F). Together, a proper *G*_min_ is required for P_II_N to realize tristability.

### Timescale of the NFL markedly influences the tristability of P_II_N circuits

It is well known that coupling an NFL with a PFL can also induce oscillation via Hopf bifurcation under some conditions^25^,^26^. For P_II_N circuits, the necessary condition for Hopf bifurcation at a steady state represented by *x* is (see *SI Text* 3):


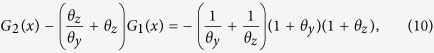


where *θ*_*y*_ and *θ*_*z*_ determine the timescales of *Y* and *Z* dynamics. Thus, both the gains and timescales have a role in governing the stability of steady states. It is worthy to explore how *θ*_*y*_ and *θ*_*z*_ affect the tristability of systems with a given gain. Take System 4 with *R* = −0.02 as an example.

**Figure 8:**
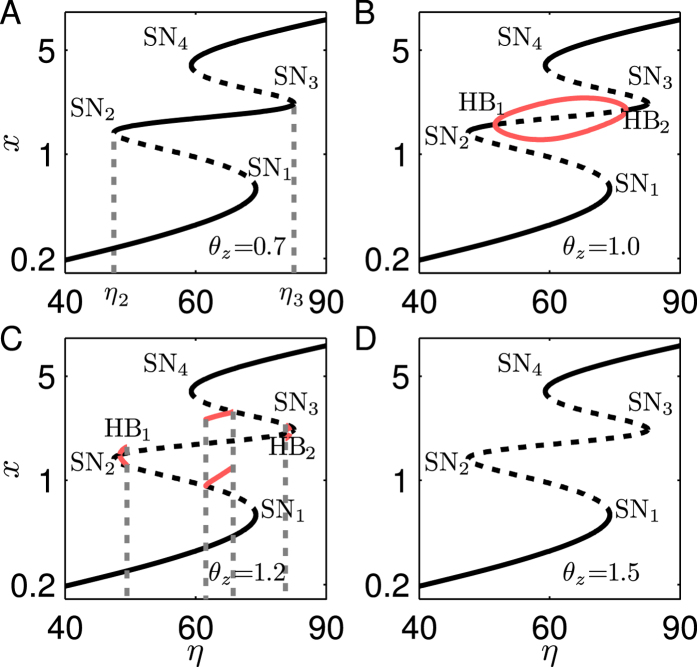
Bifurcation diagrams of P_II_N (System 4) with *R* = −0.02, *θ*_*y*_ = 1.0 and different *θ*_*z*_. (**A**) All the steady states in the intermediate branch between SN_2_ and SN_3_ are stable at *θ*_*z*_ = 0.7. (**B**) The steady states between two Hopf bifurcation points HB_1_ and HB_2_ are unstable and accompanied by stable limit cycles at *θ*_*z*_ = 1.0. Red curves denote the maximum and minimum of *x* in limit cycles. (**C**) The *η* range allowing for limit cycles is shortend at *θ*_*z*_ = 1.2. Dashed lines denote the supercritical saddle homoclinic orbit bifurcations. (**D**) All the steady states between SN_2_ and SN_3_ are unstable, and no limit cycle occurs at *θ*_*z*_ = 1.5. Both axes are in log scale.

We first fix *θ*_*y*_ at 1.0 and investigate the influence of *θ*_*z*_. The system can admit tristability at *θ*_*z*_ = 0.7 (Fig. 8A). All the steady states in the intermediate branch between SN_2_ and SN_3_ are stable. When *θ*_*z*_ rises to 1.0, two Hopf bifurcations (HB_1_ and HB_2_) appear at the intermediate branch. Thus, the steady states bounded by HB_1_ and HB_2_ become unstable, and stable limit-cycle oscillations may arise (Fig. 8B). If *θ*_*z*_ further rises to 1.2, some limit cycles disappear after a supercritical saddle homoclinic orbit bifurcation, where a limit cycle collides with a saddle and then becomes a homoclinic orbit to the saddle (Fig. 8C). If *θ*_*z*_ is increased to 1.5, Hopf bifurcation points no longer exist after the collision with SN bifurcation points (via Bogdanov-Takens (BT) bifurcation); no limit cycles appear and all the steady states in the branch between SN_2_ and SN_3_ become unstable (Fig. 8D), leaving only two stable branches. Consequently, only monostability or bistability can be realized. This kind of bistability has been reported^27^, where a bistable system exhibits diverse and flexible switching behaviors.

**Figure 9:**
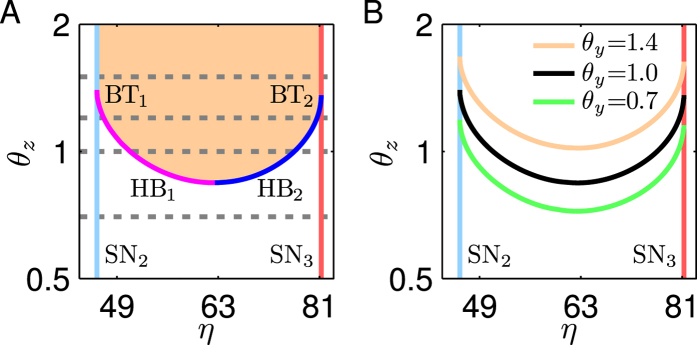
Phase diagrams of *θ*_*z*_ versus *η*. (**A**) Stability of steady states in the intermediate branch between SN_2_ and SN_3_ shown in Fig. 8 for different *θ*_*z*_. The solid curves denote the loci of Hopf bifurcation points HB_1_ (magenta) and HB_2_ (blue), and SN bifurcation points SN_2_ (cyan) and SN_3_ (red). Bogdanov-Takens bifurcations occur at the intersections between the HB and SN loci, BT_1_ and BT_2_. Four dashed lines denote the values of *θ*_*z*_ corresponding to the bifurcation diagrams in Fig. 8. (**B**) The loci of Hopf bifurcation points for differnt *θ*_*y*_. Both axes are in log scale.

To systematically demonstrate how *θ*_*z*_ influences the stability of steady states in the intermediate branch between SN_2_ and SN_3_, we then construct a phase diagram of *θ*_*z*_ versus *η*, where four loci of SN_2_, SN_3_, HB_1_ and HB_2_ are traced out (Fig. 9A). The loci of HB_1_ and HB_2_ meet at *θ*_*z*_ = 0.84, the minimum of *θ*_*z*_ that allows for Hopf bifurcation (with *η* = 62.53). The domains below and above these two loci correspond to the stability and instability of steady states, respectively. Therefore, some steady states between SN_2_ and SN_3_ become unstable if *θ*_*z*_ > 0.84. For *θ*_*z*_ > 1.36 (the value at which HB_2_ and SN_3_ meet and a BT bifurcation occurs), all the steady states are unstable, and tristability is completely forbidden. Conversely, all the steady states between SN_2_ and SN_3_ are stable for *θ*_*z*_ < 0.84. Thus, the system admits tristability in a wide range of *η*. Together, decreasing *θ*_*z*_ facilitates the occurrence of tristability. In other words, when the PFL is coupled with a fast enough NFL, tristability is more easily produced.

Finally, we investigate the effect of *θ*_*y*_ on the tristability. We change *θ*_*y*_ and determine the loci of Hopf bifurcation points. When increasing or decreasing *θ*_*y*_, the loci have similar shapes (Fig. 9B), and thus the above conclusion qualitatively holds true for different *θ*_*y*_. Nevertheless, when *θ*_*y*_ drops (e.g., to 0.7), the minimum of *θ*_*z*_ that allows Hopf bifurcation becomes smaller, meaning that to widen the tristability region, the NFL dynamics need to be faster. Conversely, if *θ*_*y*_ rises (e.g., to 1.4), the requirement for a fast NFL is loosened. Thus, *θ*_*y*_ can influence the timescale range of the NFL required for tristability.

### Conditions for different bifurcation diagrams of simplified P_II_N systems

To further interpret the above results, we approximate the gain functions by piecewise linear functions and derive the conditions for different types of bifurcation diagrams (see *SI Text* 6). Set 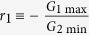, 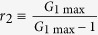, 
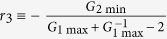
, 
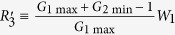
, 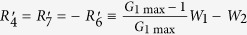, and 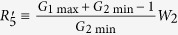. According to Table 1, we can explain how the features of individual feedback loops affect the tristability in P_II_N circuits. First, the bifurcation diagrams of type IV-VII appear only when *W*_1_/*W*_2_ > *r*_1_ for *G*_1max_ + *G*_2min_ > 1 or *W*_1_/*W*_2_ > *r*_3_ for *G*_1max_ + *G*_2min_ < 1. Obviously, *r*_1_ > 1 for *G*_1max_ + *G*_2min_ > 1 and *r*_3_ > *r*_2_ > 1 for *G*_1max_ + *G*_2min_ < 1 always hold. Thus, *W*_2_ < *W*_1_ is necessary for tristability (Fig. S4). Second, a limited range of |*R*| is also crucial for tristability. For *G*_1max_ + *G*_2min_ < 1, tristability can be effectively induced when *R*~0; for *G*_1max_ + *G*_2min_ > 1, a non-zero but moderate *R* is required (Fig. 7). Third, *G*_1max_ or *G*_2min_ influences the critical values of *W*_1_/*W*_2_ and |*R*| for tristability. With *G*_1max_, *W*_1_ and *W*_2_ fixed, for example, decreasing *G*_2min_ can increase *r*_3_ but decrease *r*_1_. Given *G*_1max_ + *G*_2min_ > 1, a decrease in *G*_2min_ can lead to a change from *W*_1_/*W*_2_ < *r*_1_ to *W*_1_/*W*_2_ > *r*_1_ and the occurrence of tristability. Given *G*_1max_ + *G*_2min_ < 1, however, a reduction in *G*_2min_ can cause a change from *W*_1_/*W*_2_ > *r*_3_ to *W*_1_/*W*_2_ < *r*_3_, thus eliminating the tristability at any *R*. This accounts for why a proper *G*_2min_ is required for inducing tristability. Finally, it is worthy to note that the estimates of critical thresholds in Table 1 would be in more agreement with numerical results when a more accurate approximation to the gain functions is acquired.

### Dynamics in tristable systems

It is worth noting that the tristable P_II_N and P_II_P_II_ circuits may exhibit distinct dynamics in approaching a steady state. Take System 4 with *R* = −0.02 and System 1 with *R* = 2.04 as examples of the P_II_N and P_II_P_II_ circuits, respectively; they exhibit tristability separately at *η* = 66 (Fig. S7A) and *η* = 250 (Fig. S8A). We plot the phase portraits to demonstrate their dynamics around each steady state. When approaching the intermediate stable steady state, the P_II_N system shows damped oscillation (Fig. S7B), whereas the P_II_P_II_ system does not (Fig. S8B). This difference is related to distinct features of eigenvalues for that state. For the P_II_N system, the real part of a pair of conjugate complex eigenvalues is greater than the real eigenvalue (Fig. S7C,D); thus, the oscillatory mode decays more slowly, resulting in damped oscillation. In contrast, a real eigenvalue has the maximal negative real part for the P_II_P_II_ system (Fig. S8C,D). Taken together, such a dynamic difference may be used to distinguish between the two types of tristable systems.

## Discussion

We revealed how the features of individual feedback loops affect the presence of tristability in the coupled system and determined various conditions for tristability to arise in terms of logarithmic gain. For two coupled bistable PFLs, there should be a moderate difference between their activation thresholds. When coupled with a bistable PFL, an NFL should have a narrower gain curve and a comparable activation threshold compared with the PFL, a proper minimum gain and fast dynamics. These results have important implications for realizing tristability in interlinked feedback loops.

The current work indicates that the logarithmic gain is an important measure, greatly facilitating the identification of critical features of feedback loops that contribute to the tristability. This is because the gain is the derivative of the transfer function, providing sufficient information about its nonlinearity. With this gain, it is easy to judge whether a system satisfies the essential condition for tristability. One feasible method to experimentally measure the gain is to interrupt a feedback loop and characterize the input-output relationship of the open-loop system^21^,^23^. Our theoretical framework can also be applicable to the other two types of dual-loop circuits shown in Fig. 1A,B, given two loops are multiplicatively coupled (see *SI Text* 7). In this scenario, the additivity of logarithmic gains and the necessary condition for saddle-node bifurcation still hold true. If the gain curves of individual loops are also bell shaped, the conditions for tristability in the coupled loops can be analyzed similarly.

Analyzing the gain of individual feedback loops may gain insight into the feature of coupled feedback loops. For example, the system comprising the NR2F2/OCT4 and OCT4/miR-302/NR2F2 PFLs governs a one-step transition from the undifferentiated to differentiated state^18^ (Fig. 1D). Intuitively, this system may not exhibit tristability. As seen in Fig. 2F, however, the bifurcation diagram of type VII, admitting a one-step transition (given that there is no critical slowing down), also contains a tristability region. Thus, the system may possess a hidden intermediate state that is hard to observe. This awaits experimental verification, which may begin with testing whether both the two PFLs exhibit bistability (equivalent to checking whether *G*_1max_ and *G*_2max_ exceed 1.0).

The epithelial-mesenchymal transition (EMT) has been widely studied because it is involved in various processes such as cancer metastasis, wound repair and embryonic development^28^. Two theoretical models revealed how the tristability and two-step transitions underlie the EMT^4^,^5^. Despite differences between the two models in modeling details, the tristability is realized in a dual-PFL module, and the two-step transitions are governed by the sequential activation of two PFLs. Moreover, different parameter values lead to distinct transition routes^5^,^6^. Indeed, here we identify four types of bifurcation diagrams with a tristability region, and they admit distinct routes of transitions among multiple stable states (Fig. 2). More importantly, distinct transitions can be guaranteed by adjusting the features of individual feedback loops. This provides an effective method for interlinking two PFLs to accomplish the expected transition, which may be applicable to controllable cell differentiation and reprogramming.

Very recently, it was shown that the circuit comprising the Ovol2/Zeb1, Snail1/miR-34a and Zeb1/miR-200 PFLs can realize tetra-stability in the EMT^7^. Of note, there exists another PFL involving an indirect regulation, Ovol2/TGF-*β*/Snail1/Zeb1, which can be coupled with the Ovol2/Zeb1 loop in a similar manner to that in Fig. 1C. According to our results, this coupled system may also exhibit tristability over some range of parameters even when the Snail1/miR-34a and Zeb1/miR-200 PFLs are blocked, which awaits further justification.

Whereas it was widely shown that coupling a PFL with an NFL can act as a bistable switch, an oscillator or excitable device^16^,^29^,^30^,^31^,^32^,^33^ and also improve the robustness of those functional motifs^34^,^35^,^36^,^37^, whether it can generate tristability remains open. Here, we found that the coupled system can admit tristability when the NFL quenches the PFL locally in terms of the gain: the single bell in the *G*(*x*) curve is split into two, equivalent to production of a dual-PFL system. The timescale of the NFL should be fast enough; otherwise, the coupled system may exhibit oscillation or bistability^26^,^27^. As far as we know, there is no experimental observation supporting such a novel tristable switch; it would be interesting to experimentally validate this two-step transition.

The current work is limited to deterministic analysis. Given the inherent stochasticity in biological systems, the robustness of multiple stable states against intrinsic fluctuation is important for their existence in a noisy environment. Moreover, the robustness to extrinsic signal fluctuation is also critical for multi-stable switches to respond accurately. Thus, it is worth exploring the influence of intrinsic and extrinsic noise on the presence of and transition between multiple steady states.

## Additional Information

**How to cite this article**: Huang, B. *et al*. Realization of tristability in a multiplicatively coupled dual-loop genetic network. *Sci. Rep.* **6**, 28096; doi: 10.1038/srep28096 (2016).

## Supplementary Material

Supplementary Information

## Figures and Tables

**Table 1:** Conditions for different types of bifurcation diagrams that simplified P_II_N systems (with *G*_1max_ > 1) can admit.

G_1max_ + G_2min_	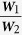	|*R*|	type
(1, +∞)	(0, *r*_1_)	[0, +∞)	II
	(*r*_1_, +∞)	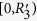	II
		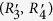	IV–VII
		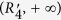	II
(−∞, 1)	(0, *r*_1_)	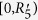	I
		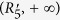	II
	(*r*_1_, *r*_2_)	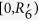	I
		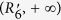	II
	(*r*_2_, *r*_3_)	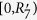	III
		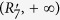	II
	(*r*_3_, +∞)	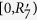	IV–VII
		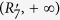	II
