# Supplementary material for: Realization of tristability in a multiplicatively coupled dual-loop genetic network
Source: Sci Rep. 2016 Jul 5;6:28096. doi: 10.1038/srep28096 (PMC4932522; doi:10.1038/srep28096)
Supplement: Supplementary Information [file srep28096-s1.pdf]

Supplementary Information

Realization of tristability in a multiplicatively coupled dual-loop genetic network

Bo Huang, Yun Xia, Feng Liu, and Wei Wang

This document includes 1) Supplementary Text describing the modeling details, various derivations, and numerical method, 2) eight Supplementary Figures, and 3) three Supplementary Tables.

I. SUPPLEMENTARY TEXT

1. DERIVATION OF THE DIMENSIONLESS EQUATIONS FOR THE MODEL

Here, we present a detailed derivation of the dimensionless differential equations. The model comprises two transcription factors (TFs) and one microRNA (miRNA), constituting two interlinked feedback loops.

1.1 Transcriptional dynamics

The transcription process is characterized by a simple two-state model—the promoter of a gene can be bound by n TFs or unbound. We assume that the transitions between unbound (U) and bound (B) states of the promoter are both a one-step process:

$$n\mathrm{TF} + U \rightleftarrows B. \tag{1}$$

According to the law of mass action, the rates of forward and backward reactions are

$$\begin{aligned} k_{U\to B} &= k_B[\mathrm{TF}]^n, \\ k_{B\to U} &= k_U, \end{aligned}$$

where [TF] is the concentration of TFs, and k_B and k_U denote the rate constants associated with binding and dissociating, respectively. The evolution of the probability for the promoter bound by TFs, P_B, is governed by the following chemical master equation:

$$\frac{dP_B(t)}{dt} = k_{U\to B}P_U(t) - k_{B\to U}P_B(t), \tag{2}$$

where P_U is the probability of the promoter unbound by TFs, i.e., $P_B + P_U = 1$. The stationary distributions for the unbound and bound states of the promoter obey

$$P_U^{\mathrm{ss}} = \frac{k_U}{k_B[\mathrm{TF}]^n + k_U}, \tag{3}$$

$$P_B^{\mathrm{ss}} = \frac{k_B[\mathrm{TF}]^n}{k_B[\mathrm{TF}]^n + k_U}. \tag{4}$$

Two different states of the promoter allow for different rates of transcription conducted by RNA polymerase. k_0 and k_1 denote the transcription rate when the promoter is unbound or bound, respectively. Given the binding and dissociating rates of TFs are much faster than the elongation rate, the average transcription rate $\bar{k}$ obeys

$$\begin{aligned} \bar{k}([\mathrm{TF}]) &= k_0P_U + k_1P_B \\ &= \frac{k_0k_U + k_1k_B[\mathrm{TF}]^n}{k_B[\mathrm{TF}]^n + k_U} \\ &= \frac{k_0 + k_1\left(\frac{[\mathrm{TF}]}{K}\right)^n}{1 + \left(\frac{[\mathrm{TF}]}{K}\right)^n}, \end{aligned} \tag{5}$$

with $K = \sqrt[n]{\frac{k_U}{k_B}}$ describing the binding affinity of TF to the promoter. $k_1 > k_0$ when TF activates transcription, whereas $k_1 < k_0$ when TF inhibits transcription. If transcription is initialized only after TF binds to the promoter, k_0 should be zero, leading to

$$\bar{k}([\mathrm{TF}]) = \frac{k_1 \left(\frac{[\mathrm{TF}]}{K}\right)^n}{1 + \left(\frac{[\mathrm{TF}]}{K}\right)^n}. \tag{6}$$

If transcription is completely prevented after TF binds to the promoter, k_1 should be zero, resulting in

$$\bar{k}([\mathrm{TF}]) = \frac{k_0}{1 + \left(\frac{[\mathrm{TF}]}{K}\right)^n}. \tag{7}$$

Of note, the Hill coefficient is not necessarily a positive integer. The above formulas were widely used to describe the transcription dynamics [1, 2].

1.2 Dynamics of microRNA-mediated translational repression

miRNA is a class of short non-coding RNAs with about 22 nucleotides. It can function as a probe to detect the target mRNA by fully or partially binding to its specific sequences in 3' UTR. Meanwhile, as part of miRNA-induced silencing complex (miRISC), miRNA can lead the miRISC to deal with its target mRNA by degrading it or preventing it from being translated. No matter how miRISC processes mRNA, the subsequent translation of mRNA will be prevented, which is equivalent to a decrease in the effective amount of mRNA. Thus, we consider the process of regulation by miRNA as the following reaction:

$$M + m \longrightarrow M.$$

miRNA actually functions as an enzyme during its lifetime. Instead of describing this reaction by the Michaelis-Menten kinetics, we take it as a second-order reaction for simplicity. Thus, the reaction rate is $k_{\mathrm{Mm}}[M][m]$.

1.3 Equations for the model

Combining the above two processes, we write down the equations governing the dynamics of the circuit:

$$\frac{d[m_1]}{dt} = \frac{k_{m_1}}{1 + \left(\frac{[Y]}{K_X}\right)^{n_1}} - \gamma_{m_1}[m_1] - k_{\mathrm{Zm}_1}[Z][m_1], \tag{8}$$

$$\frac{d[X]}{dt} = \beta_X[m_1] - \gamma_X[X], \tag{9}$$

$$\frac{d[m_2]}{dt} = \frac{k_{m_2}}{1 + \left(\frac{[X]}{K_Y}\right)^{n_2}} - \gamma_{m_2}[m_2], \tag{10}$$

$$\frac{d[Y]}{dt} = \beta_Y[m_2] - \gamma_Y[Y], \tag{11}$$

$$\frac{d[Z]}{dt} = \frac{k_{Z0} + k_{Z1}\left(\frac{[Y]}{K_Z}\right)^{n_3}}{1 + \left(\frac{[Y]}{K_Z}\right)^{n_3}} - \gamma_Z[Z], \tag{12}$$

where $[m_1]$, $[m_2]$, $[X]$, $[Y]$, and $[Z]$ denote the concentrations of X mRNA, Y mRNA, X protein, Y protein, and miRNA Z.

Given that the lifetime of mRNA is much shorter than those of proteins [3] and miRNA [4], the amount of mRNA

can rapidly reach an equilibrium. Thus, the right-hand sides of Eqs. (8) and (10) are set to zero, leading to

$$[m_1] = \frac{k_{m_1}}{\left[1+\left(\frac{[Y]}{K_X}\right)^{n_1}\right](\gamma_{m_1}+k_{\mathrm{Zm}_1}[Z])}, \tag{13}$$

$$[m_2] = \frac{k_{m_2}}{\left[1+\left(\frac{[X]}{K_Y}\right)^{n_2}\right]\gamma_{m_2}}. \tag{14}$$

Consequently, we obtain the following equations

$$\frac{d[X]}{dt} = \frac{k_X}{\left[1+\left(\frac{[Y]}{K_X}\right)^{n_1}\right](1+k_Z[Z])} - \gamma_X[X], \tag{15}$$

$$\frac{d[Y]}{dt} = \frac{k_Y}{1+\left(\frac{[X]}{K_Y}\right)^{n_2}} - \gamma_Y[Y], \tag{16}$$

$$\frac{d[Z]}{dt} = \frac{k_{Z0}+k_{Z1}\left(\frac{[Y]}{K_Z}\right)^{n_3}}{1+\left(\frac{[Y]}{K_Z}\right)^{n_3}} - \gamma_Z[Z], \tag{17}$$

where $k_X = \frac{k_{m_1}\beta_X}{\gamma_{m_1}}$ and $k_Y = \frac{k_{m_2}\beta_Y}{\gamma_{m_2}}$ denote the effective production rates of X and Y, respectively, and $k_Z = \frac{k_{\mathrm{Zm}_1}}{\gamma_{m_1}}$ represents the effective strength of inhibition of Y mRNA translation.

The dimensionless time, variables and parameters are defined as follows: $\tau = \gamma_X t$, $\theta_y = \frac{\gamma_X}{\gamma_Y}$, $\theta_z = \frac{\gamma_X}{\gamma_Z}$, $x = \frac{[X]}{K_Y}$, $y = \frac{[Y]}{K_X}$, $z = \frac{[Z]}{k_{Z0}/\gamma_Z}$, $\eta = \frac{k_X/\gamma_X}{K_Y}$, $\kappa = \frac{k_Y/\gamma_Y}{K_X}$, $\alpha = \frac{k_{Z1}}{k_{Z0}}$, $K = \frac{K_Z}{K_X}$, and $\delta = k_Z\frac{k_{Z0}}{\gamma_Z}$. The equations in dimensionless forms are:

$$\frac{dx}{d\tau} = \frac{\eta}{(1+y^{n_1})(1+\delta z)} - x, \tag{18}$$

$$\theta_y\frac{dy}{d\tau} = \frac{\kappa}{1+x^{n_2}} - y, \tag{19}$$

$$\theta_z\frac{dz}{d\tau} = \frac{1+\alpha\left(\frac{y}{K}\right)^{n_3}}{1+\left(\frac{y}{K}\right)^{n_3}} - z. \tag{20}$$

Similarly, the dimensionless equations for the TF-TF and TF-M-TF loops are separately

$$\frac{dx}{d\tau} = \frac{\eta}{1+y^{n_1}} - x, \tag{21}$$

$$\theta_y\frac{dy}{d\tau} = \frac{\kappa}{1+x^{n_2}} - y, \tag{22}$$

and

$$\frac{dx}{d\tau} = \frac{\eta}{1+\delta z} - x, \tag{23}$$

$$\theta_y\frac{dy}{d\tau} = \frac{\kappa}{1+x^{n_2}} - y, \tag{24}$$

$$\theta_z\frac{dz}{d\tau} = \frac{1+\alpha\left(\frac{y}{K}\right)^{n_3}}{1+\left(\frac{y}{K}\right)^{n_3}} - z. \tag{25}$$

2. LINEAR STABILITY ANALYSIS AND BIFURCATION ANALYSIS

In this section, we analyze the linear stability of the systems of individual and interlinked feedback loops at steady states. We also give the necessary conditions for saddle-node (SN) bifurcation and Hopf bifurcation.

2.1 Linear stability analysis

Set $f(y) = \frac{1}{1+y^{n_1}}$, $g(z) = \frac{1}{1+\delta z}$, $h(x) = \frac{\kappa}{1+x^{n_2}}$, and $u(y) = \frac{1+\alpha\left(\frac{y}{K}\right)^{n_3}}{1+\left(\frac{y}{K}\right)^{n_3}}$. η is chosen as the control parameter of the system. According to Eqs. (18)–(20), the steady-state equations for the coupled system are:

$$x = \eta f(y)g(z), \tag{26}$$
$$y = h(x), \tag{27}$$
$$z = u(y). \tag{28}$$

The steady-state values of x, y and z for a given η are denoted by x_s, y_s and z_s. Note that these equations can be reduced to the following single equation:

$$x = \eta[f \circ h(x)][g \circ u \circ h(x)], \tag{29}$$

where the symbol $\circ$ stands for the composition of functions. Obviously, the solution to this equation is the steady-state value of x, i.e., x_s. Moreover, given any $x_s \in [0, +\infty)$, the corresponding η, y_s and z_s can be calculated from Eqs. (29), (27) and (28), respectively. Thus, we can use $x_s \in [0, +\infty)$ to represent any steady state for different η. In the following, the subscript of x_s is omitted and x stands for the steady-state value of X.

To determine the linear stability of the system at steady states, we investigate the roots of the characteristic equation of the Jacobian matrix associated with the ordinary differential equations evaluated at the steady states. For the coupled system, the characteristic equation for Eqs. (18)–(20) is

$$\lambda^3 + A_{\mathrm{C}}\lambda^2 + B_{\mathrm{C}}\lambda + C_{\mathrm{C}} = 0, \tag{30}$$

where

$$A_{\mathrm{C}} = \frac{1}{\theta_y} + \frac{1}{\theta_z} + 1,$$
$$B_{\mathrm{C}} = \frac{1}{\theta_y}\{1 - \frac{x}{f \circ h(x)}\frac{d}{dx}[f \circ h(x)]\} + \frac{1}{\theta_z} + \frac{1}{\theta_y\theta_z},$$
$$C_{\mathrm{C}} = \frac{1}{\theta_y\theta_z}\{1 - \frac{x}{f \circ h(x)}\frac{d}{dx}[f \circ h(x)] - \frac{x}{g \circ u \circ h(x)}\frac{d}{dx}[g \circ u \circ h(x)]\}.$$

Note that η is replaced with the function of x according to Eq. (29), i.e., $\eta = x/\{[f \circ h(x)][g \circ u \circ h(x)]\}$. Thus, all the above coefficients only depend on x. If all roots of Eq. (30) have a negative real part, the steady state is stable. If any root has a positive real part, the steady state is unstable.

Similarly, the characteristic equation for the TF-TF loop is

$$\lambda^2 + B_1\lambda + C_1 = 0, \tag{31}$$

with

$$B_1 = \frac{1}{\theta_y} + 1,$$
$$C_1 = \frac{1}{\theta_y}\{1 - \frac{x}{f \circ h(x)}\frac{d}{dx}[f \circ h(x)]\}.$$

The characteristic equation for the TF-M-TF loop is

$$\lambda^3 + A_2\lambda^2 + B_2\lambda + C_2 = 0, \tag{32}$$

with

$$A_2 = \frac{1}{\theta_y} + \frac{1}{\theta_z} + 1,$$
$$B_2 = \frac{1}{\theta_y} + \frac{1}{\theta_z} + \frac{1}{\theta_y\theta_z},$$
$$C_2 = \frac{1}{\theta_y\theta_z}\{1 - \frac{x}{g \circ u \circ h(x)}\frac{d}{dx}[g \circ u \circ h(x)]\}.$$

2.2 Bifurcation analysis

For SN bifurcation to occur, the characteristic equation must have a root $\lambda = 0$. That is, the constant term of the characteristic equation should be zero. For the coupled system, the necessary condition is

$$\frac{x}{f\circ h(x)}\frac{d}{dx}[f\circ h(x)] + \frac{x}{g\circ u\circ h(x)}\frac{d}{dx}[g\circ u\circ h(x)] \;=\; 1. \tag{33}$$

For the TF-TF and TF-M-TF loops, the necessary conditions are separately

$$\frac{x}{f\circ h(x)}\frac{d}{dx}[f\circ h(x)] \;=\; 1, \tag{34}$$

and

$$\frac{x}{g\circ u\circ h(x)}\frac{d}{dx}[g\circ u\circ h(x)] \;=\; 1. \tag{35}$$

For Hopf bifurcation to occur, the characteristic equation must have a pair of pure imaginary roots $\lambda = \pm i\omega$, where ω is a positive real number. For the coupled system, the necessary condition is

$$\begin{aligned}&\frac{x}{g\circ u\circ h(x)}\frac{d}{dx}[g\circ u\circ h(x)] - \left(\frac{\theta_z}{\theta_y}+\theta_z\right)\frac{x}{f\circ h(x)}\frac{d}{dx}[f\circ h(x)]\\ &= -\left(\frac{1}{\theta_y}+\frac{1}{\theta_z}\right)(1+\theta_y)(1+\theta_z).\end{aligned} \tag{36}$$

For the TF-M-TF loop, the necessary condition is

$$\frac{x}{g\circ u\circ h(x)}\frac{d}{dx}[g\circ u\circ h(x)] \;=\; -\left(\frac{1}{\theta_y}+\frac{1}{\theta_z}\right)(1+\theta_y)(1+\theta_z). \tag{37}$$

Because the TF-TF loop is always a PFL, Hopf bifurcation is impossible. From Eqs. (36) and (37), the occurrence of Hopf bifurcation depends on θ_y and θ_z. By properly adjusting θ_y and θ_z, the condition for Hopf bifurcation can be never satisfied for any x.

3. LOGARITHMIC GAIN

In this section, we introduce the logarithmic gain of the systems of individual and coupled feedback loops based on several previous studies [5–8]. A single feedback loop can sometimes be regarded as self-regulation of a specific component via other components. Consider a feedback loop composed of N components $C_i (i = 1, 2, \cdots, N)$, each of which can only regulate its adjacent downstream component. The dimensionless concentration of C_i is denoted by $c_i (i = 1, 2, \cdots, N)$ and the strictly monotonic function describing the regulation of C_{i+1} by C_i is denoted by F_i with $i = 1, 2, \cdots, N-1$ (that of C_1 by C_N is denoted by F_N). We take this feedback loop as the self-regulation of C_1 via the other components. Assume the dynamics of this feedback loop are governed by the following ordinary differential equations:

$$\begin{aligned}\frac{dc_1}{d\tau} &= \eta F_N(c_N) - c_1,\\ \theta_{i+1}\frac{dc_{i+1}}{d\tau} &= F_i(c_i) - c_{i+1}, i = 1, 2, \cdots, N-1,\end{aligned} \tag{38}$$

where η is the control parameter and $\frac{1}{\theta_i}$ is the degradation rate of C_i normalized to that of C_1. At steady states, all the derivatives equal zero, thus yielding the following N algebraic equations:

$$\begin{aligned}c_1 &= \eta F_N(c_N),\\ c_{i+1} &= F_i(c_i), i = 1, 2, \cdots, N-1.\end{aligned} \tag{39}$$

By eliminating $c_i (i = 2, \cdots, N-1)$, we have the equation for c_1:

$$c_1 \;=\; T(c_1), \tag{40}$$

where $T = \eta F_N \circ F_{N-1} \circ \cdots \circ F_1$. Of note, the function T is the composition of functions, each describing one of regulatory interactions in the feedback loop, and the way of composition reflects the propagation of these interactions. Thus, the function T indicates how C_1 regulates itself via the feedback loop. In this sense, we term T a transfer function. Owing to the strict monotonicity of F_i, T is a strictly monotonically increasing function for PFLs, or a strictly monotonically decreasing function for NFLs.

We then define the logarithmic gain (G, called gain for short) as the derivative of the transfer function T with respect to c_1 in log-log coordinates:

$$\begin{aligned} G(c_1) &= \frac{d \ln T(c_1)}{d \ln c_1} \\ &= \frac{c_1}{(F_N \circ F_{N-1} \circ \cdots \circ F_1)(c_1)} \frac{d(F_N \circ F_{N-1} \circ \cdots \circ F_1)(c_1)}{dc_1}. \end{aligned} \tag{41}$$

Because of the strict monotonicity of T, G is always positive for PFLs or negative for NFLs.

To formulate the necessary condition for SN bifurcation, we calculate the determinant of the system's Jacobian matrix:

$$\det(J_{F_1, F_2, \cdots, F_N}(c_1, c_2, \cdots, c_N)) = \frac{(-1)^N}{\prod_{i=2}^{N} \theta_i} \left(1 - \eta \prod_{i=1}^{N} \frac{dF_i(c_i)}{dc_i} \right), \tag{42}$$

where $c_i (i = 1, 2, \cdots, N)$ satisfy Eq. (39). For SN bifurcation to occur, the determinant must be zero, thus yielding $\eta \prod_{i=1}^{N} \frac{dF_i(c_i)}{dc_i} = 1$. According to the chain rule, this equation can be rewritten as

$$G(c_1) = 1. \tag{43}$$

This is just the necessary condition for SN bifurcation in terms of the gain.

Based on the above definitions, the transfer function and gain of the TF-TF loop are

$$T_1(x) = (\eta f \circ h)(x), \tag{44}$$

and

$$G_1(x) = \frac{x}{f \circ h(x)} \frac{d}{dx} [f \circ h(x)], \tag{45}$$

respectively. For the TF-M-TF loop, the transfer function and gain are

$$T_2 = (\eta g \circ u \circ h)(x), \tag{46}$$

and

$$G_2(x) = \frac{x}{g \circ u \circ h(x)} \frac{d}{dx} [g \circ u \circ h(x)]. \tag{47}$$

Similarly, we can calculate the transfer function and gain of coupled feedback loops as follows:

$$T_{\mathrm{C}}(x) = [\eta (f \circ h)(g \circ u \circ h)](x), \tag{48}$$

and

$$G_{\mathrm{C}}(x) = \frac{x}{f \circ h(x)} \frac{d}{dx} [f \circ h(x)] + \frac{x}{g \circ u \circ h(x)} \frac{d}{dx} [g \circ u \circ h(x)]. \tag{49}$$

Notably,

$$G_{\mathrm{C}}(x) = G_1(x) + G_2(x), \tag{50}$$

i.e., the gain of coupled feedback loops is just the sum of those of individual feedback loops.

The necessary condition for Hopf bifurcation in the coupled system in terms of gains is

$$G_2(x) - \left(\frac{\theta_z}{\theta_y} + \theta_z \right) G_1(x) = - \left(\frac{1}{\theta_y} + \frac{1}{\theta_z} \right) (1 + \theta_y)(1 + \theta_z), \tag{51}$$

which also depends on the timescales. For the TF-M-TF loop, the necessary condition is

$$G_2(x) = - \left(\frac{1}{\theta_y} + \frac{1}{\theta_z} \right) (1 + \theta_y)(1 + \theta_z). \tag{52}$$

4. ESTIMATION OF THE WIDTH OF THE BISTABILITY REGION AND THRESHOLDS OF R FOR TRISTABILITY IN A DUAL-PFL SYSTEM

4.1 Estimation of the width of the bistability region for a single PFL

In this section, we estimate the width of a bistability region (W_{B}) for a single PFL as a function of the maximum gain ($G_{\max} > 1$) and the width of the effective concentration range (W). The $G_{\max}$ is defined as the global maximum of G. The W is defined as the logarithmic difference between w_2 and w_1, i.e., $W = \ln(w_2/w_1)$ ($w_2 > w_1$) with w_1 and w_2 denoting the x values at half points (i.e., $G(w_1) = G(w_2) = G_{\max}/2$). The W_{B} is defined as the logarithmic difference between η_1 and η_2, i.e., $W_{\mathrm{B}} = \ln \frac{\eta_1}{\eta_2}$, where η_1 and η_2 are the upper and lower thresholds for the bistability region, respectively. The x values at η_1 and η_2 are denoted by b_1 and b_2, respectively. According to Eq. (43), $G(b_1) = G(b_2) = 1$ (Fig. 1).

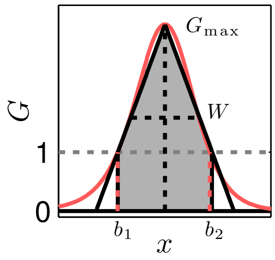

FIG. 1: Illustration of the approximation of $G(x)$. The area under the bell-shaped curve of $G(x)$ on the interval $[b_1, b_2]$ ($G(b_1) = G(b_2) = 1$) is approximated by the area of an isosceles triangle minus those of two small right-angled triangles below $G(x) = 1$, i.e., the area of the gray region. The isosceles triangle has a height of $G_{\max}$ and base length of $2W$, while the right-angled triangles have heights of 1. The x-axis is in log scale.

To explicitly present the relationship between η and x, we define the transfer function in units of η, $\tilde{T}$:

$$\tilde{T}(x) = \frac{T(x)}{\eta}. \tag{53}$$

Thus,

$$\eta = \frac{x}{\tilde{T}(x)}. \tag{54}$$

The width of the bistability region can be expressed as

$$\begin{aligned} W_{\mathrm{B}} &= \ln \frac{b_1}{b_2} \frac{\tilde{T}(b_2)}{\tilde{T}(b_1)} \\ &= [\ln \tilde{T}(b_2) - \ln \tilde{T}(b_1)] - (\ln b_2 - \ln b_1). \end{aligned} \tag{55}$$

G can be expressed in terms of $\tilde{T}$:

$$G(x) = \frac{d \ln \tilde{T}(x)}{d \ln x}, \tag{56}$$

which is equivalent to the expression of G in terms of $T(x)$. Thus,

$$W_{\mathrm{B}} = \int_{b_1}^{b_2} G(x) d \ln x - (\ln b_2 - \ln b_1), \tag{57}$$

That is, W_{B} only depends on the part of $G(x) \geqslant 1$.

To estimate the integral and $(\ln b_2 - \ln b_1)$, we use an isosceles triangle to replace the bell-shaped part of $G(x)$. This triangle has a height of $G_{\max}$ and a base length of $2W$ (Fig. 1). Then, the integral is approximately

$$\int_{b_1}^{b_2} G(x) d \ln x \approx W G_{\max} [1 - \frac{1}{G_{\max}^2}]. \tag{58}$$

It is easy to see

$$\frac{\ln b_2 - \ln b_1}{2W} \approx \frac{G_{\max} - 1}{G_{\max}}. \tag{59}$$

Substituting Eqs. (58) and (59) into Eq. (57), we have the following estimate:

$$W_{\mathrm{B}} \approx W(G_{\max} + \frac{1}{G_{\max}} - 2). \tag{60}$$

4.2 Estimation of the widths of two separate bistability regions for the coupled system

Similar to Eq. (57), the widths of two separate bistability regions, $W_{\mathrm{BC1}} = \ln(\eta_1/\eta_2)$ and $W_{\mathrm{BC2}} = \ln(\eta_3/\eta_4)$, can be determined by two bell-shaped parts of the $G_{\mathrm{C}}(x)$ curve (more exactly, the parts of $G_{\mathrm{C}}(x) \geqslant 1$), i.e.,

$$W_{\mathrm{BC1}} = \int_{c_{11}}^{c_{12}} G_{\mathrm{C}}(x) d\ln x - (\ln c_{12} - \ln c_{11}), \tag{61}$$

and

$$W_{\mathrm{BC2}} = \int_{c_{21}}^{c_{22}} G_{\mathrm{C}}(x) d\ln x - (\ln c_{22} - \ln c_{21}), \tag{62}$$

where c_{11}, c_{12}, c_{21} and c_{22} are the x values at η_i $(i = 1 \sim 4)$. That is, $G_{\mathrm{C}}(c_{11}) = G_{\mathrm{C}}(c_{12}) = G_{\mathrm{C}}(c_{21}) = G_{\mathrm{C}}(c_{22}) = 1$ and $c_{11} < c_{12} < c_{21} < c_{22}$. Because of the 'bandpass' feature and large R, the two bell-shaped parts of the $G_{\mathrm{C}}(x)$ curve can be approximately represented by $G_1(x)$ and $G_2(x)$, respectively, leading to

$$W_{\mathrm{BC1}} \approx \int_{b_{11}}^{b_{12}} G_1(x) d\ln x - (\ln b_{12} - \ln b_{11}), \tag{63}$$

and

$$W_{\mathrm{BC2}} \approx \int_{b_{21}}^{b_{22}} G_2(x) d\ln x - (\ln b_{22} - \ln b_{21}), \tag{64}$$

with $G_1(b_{11}) = G_1(b_{12}) = G_2(b_{21}) = G_2(b_{22}) = 1$ and $b_{11} < b_{12} < b_{21} < b_{22}$. Notably, the right-hand sides of Eqs. (63) and (64) separately equal the widths of the bistability range for two individual loops, i.e., W_{B1} and W_{B2} [cf. Eq. (57)]. Based on Eq. (60), we further have

$$W_{\mathrm{BC1}} \approx W_1(G_{1\max} + \frac{1}{G_{1\max}} - 2), \tag{65}$$

and

$$W_{\mathrm{BC2}} \approx W_2(G_{2\max} + \frac{1}{G_{2\max}} - 2). \tag{66}$$

4.3 Estimation of the width of the intermediate branch of stable steady states for coupled PFLs

Here, we estimate the width of the intermediate branch of stable steady states, W_{I}, in the case of $x_{2\mathrm{m}} > x_{1\mathrm{m}}$. Similarly,

$$W_{\mathrm{I}} = \int_{c_{21}}^{c_{12}} G_{\mathrm{C}}(x) d\ln x - (\ln c_{12} - \ln c_{21}), \tag{67}$$

where c_{12} and c_{21} ($c_{12} < c_{21}$) are the x values at the lower (η_2) and upper (η_3) thresholds for the intermediate branch, respectively. That is, $G_{\mathrm{C}}(c_{12}) = G_{\mathrm{C}}(c_{21}) = 1$. For a large R, two bell-shaped parts of the G_{C} curve can maintain the features of the G_1 and G_2 curves. Thus, these parts can be approximated by G_1 and G_2, and we have $c_{12} \approx b_{12}$ and $c_{21} \approx b_{21}$, with $G_1(b_{12}) = G_2(b_{21}) = 1$.

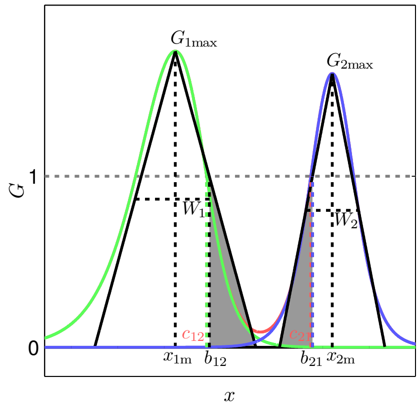

FIG. 2: Illustration of the approximation of $G_{\mathrm{C}}(x)$. Two bell-shaped parts of the $G_{\mathrm{C}}(x)$ curve are replaced with $G_1(x)$ and $G_2(x)$. $b_{12} \approx c_{12}$ and $b_{21} \approx c_{21}$, where $G_{\mathrm{C}}(c_{12}) = G_{\mathrm{C}}(c_{21}) = G_1(b_{12}) = G_2(b_{21}) = 1$. The area under two bell-shaped parts of the $G_{\mathrm{C}}(x)$ curve on the interval $[c_{12}, c_{21}]$ is approximated by the total area of two small gray right-angled triangles near the base angle. Two isosceles triangles have heights of $G_{1\max}$ and $G_{2\max}$ and base lengths of $2W_1$ and $2W_2$, respectively. The small gray right-angled triangles have heights of 1. The x-axis is in log scale.

Using the same approximation method mentioned above, we replace the bell-shaped parts of G_1 and G_2 with two isosceles triangles, respectively. These two triangles have heights of $G_{1\max}$ and $G_{2\max}$ and base lengths of $2W_1$ and $2W_2$, respectively. As shown in Fig. 2, the integral in Eq. (61) approximately equals the negative total area of two small gray triangles, i.e.,

$$\int_{c_{21}}^{c_{12}} G(x) d\ln x \approx -\left(\frac{W_1}{2G_{1\max}} + \frac{W_2}{2G_{2\max}}\right). \tag{68}$$

As for $(\ln c_{12} - \ln c_{21})$, it can be estimated by $(\ln b_{12} - \ln b_{21})$. Furthermore,

$$\ln b_{12} - \ln b_{21} = -[R - (\ln b_{12} - \ln x_{1\mathrm{m}}) - (\ln x_{2\mathrm{m}} - \ln b_{21})], \tag{69}$$

and

$$\ln b_{12} - \ln x_{1\mathrm{m}} \approx W_1 \frac{G_{1\max} - 1}{G_{1\max}}, \tag{70}$$

$$\ln x_{2\mathrm{m}} - \ln b_{21} \approx W_2 \frac{G_{2\max} - 1}{G_{2\max}}. \tag{71}$$

Thus, W_{I} is approximately

$$W_{\mathrm{I}} \approx R - W_1\left(1 - \frac{1}{2G_{1\max}}\right) - W_1\left(1 - \frac{1}{2G_{1\max}}\right). \tag{72}$$

4.4 Estimation of the thresholds of R for tristability

The widths of two separate bistability regions, W_{BC1} and W_{BC2}, can be estimated at:

$$W_{\mathrm{BC1}} \approx W_1\left(G_{1\max} + \frac{1}{G_{1\max}} - 2\right), \tag{73}$$

and

$$W_{\mathrm{BC2}} \approx W_2\left(G_{2\max} + \frac{1}{G_{2\max}} - 2\right), \tag{74}$$

respectively. If $W_{\mathrm{I}} > W_{\mathrm{BC1}} + W_{\mathrm{BC2}}$, two separate bistability regions appear in two ranges of η; if $W_{\mathrm{I}} < W_{\mathrm{BC1}} + W_{\mathrm{BC2}}$, a tristability region is admitted. Thus, when $W_{\mathrm{I}} = W_{\mathrm{BC1}} + W_{\mathrm{BC2}}$, the approximate upper threshold of R, $R_{2,\mathrm{appr}}$ is given by

$$R_{2,\mathrm{appr}} = W_1\left(G_{1\max} + \frac{1}{2G_{1\max}} - 1\right) + W_2\left(G_{2\max} + \frac{1}{2G_{2\max}} - 1\right). \tag{75}$$

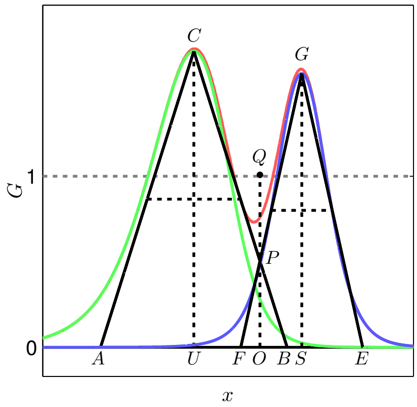

FIG. 3: Illustration for the approximation of $G_C(x)$. The approximation method for G_C is the same as that in Fig. 2. The local minimum of G_C is estimated at $|QO| = 2|PO|$, where P is the intersection of two sides of two isosceles triangles. $|CU| = G_{1max}$, $|GS| = G_{2max}$, $|UB| = W_1$, $|SF| = W_2$, and $|SU| = |R|$. The x-axis is in log scale.

If the local minimum of $G_C(x)$ between two local maxima is below 1.0, there are four SN bifurcation points, and tristability is possible; if the local minimum is elevated above 1.0, only two SN bifurcation points are admitted, and tristability is forbidden. Thus, the local minimum determines whether tristability is possible. As shown in Fig. 3, two isosceles triangles have an overlap when R is relatively small. P is the intersection of two sides of two triangles, and $|PO|$ is the distance of P to the x-axis. We set $|QO| = 2|PO|$ as the estimate of the local minimum of $G_C(x)$. Since

$$|PO| = \frac{G_{1max}}{W_1}|OB| = \frac{G_{2max}}{W_2}|OF|, \tag{76}$$

$$|BF| = W_1 + W_2 - |R|, \tag{77}$$

$$|BF| = |OF| + |OB|, \tag{78}$$

we have

$$R = W_1\left(1 - \frac{|QO|}{2G_{1max}}\right) + W_2\left(1 - \frac{|QO|}{2G_{2max}}\right). \tag{79}$$

When $|QO| = 1$, we get the approximate lower threshold of $|R|$ for tristability:

$$R_{1,appr} = W_1\left(1 - \frac{1}{2G_{1max}}\right) + W_2\left(1 - \frac{1}{2G_{2max}}\right). \tag{80}$$

5. THE NUMERICAL METHOD FOR CHANGING ONLY R

In this section, we develop a numerical method in which we only change x_{2m} while keeping G_{1max}, x_{1m}, W_1, G_{2max}, and W_2 unchanged. In this way can we merely alter $R = \ln(x_{2m}/x_{1m})$. Without altering the feature of the TF-TF loop, we only adjust two interactions: $g(z) = \frac{1}{1+\delta z}$, and $u(y) = \frac{1+\alpha\left(\frac{y}{K}\right)^{n_3}}{1+\left(\frac{y}{K}\right)^{n_3}}$, which contain four parameters. Adjusting n_3, α and K in $u(y)$ simultaneously can change x_{2m} and ensure that G_{2max} and K_2 are fixed.

First, we design two functions $F_{G_{2max}}(n_3, \alpha, K)$ and $F_{W_2}(n_3, \alpha, K)$ to numerically calculate G_{2max} and W_2 from a set of (n_3, α, K), respectively. Second, we numerically solve α and K for different n_3 from the following two equations: $F_{G_{2max}}(n_3, \alpha, K) = G_{2max}^{(T)}$ and $F_{W_2}(n_3, \alpha, K) = W_2^{(T)}$, where $G_{2max}^{(T)}$ and $W_2^{(T)}$ are the fixed values for G_{2max} and W_2, respectively. These sets of (n_3, α, K) can lead to identical G_{2max} and K_2 but different x_{2m}, thus generating distinct R.

To solve the above two equations, we adopt the following iterative method. Denote the initial values of α and K by $\alpha^{(0)}$ and $K^{(0)}$, respectively. For a given n_3, we solve the single-variable equation $F_{G_{2max}}(n_3, \alpha^{(m+1)}, K^{(m)}) - G_{2max}^{(T)} = 0$ for $\alpha^{(m+1)}$ from $m = 0$, and with this $\alpha^{(m+1)}$, we solve $F_{W_2}(n_3, \alpha^{(m+1)}, K^{(m+1)}) - W_2^{(T)} = 0$ for $K^{(m+1)}$. Then, we repeat this process and obtain two sequences $\{\alpha^{(m)}\}_{m\geqslant 0}$ and $\{K^{(m)}\}_{m\geqslant 0}$. When these two sequences converge sufficiently, we stop iterating and set the last elements in the two sequences as the values of α and K, respectively.

6. THE CONDITIONS FOR DIFFERENT TYPES OF BIFURCATION DIAGRAMS AND DERIVATION OF THE THRESHOLDS OF R FOR TRISTABILITY IN SIMPLIFIED $P_{II}N$ SYSTEMS

Here we construct several reduced $P_{II}N$ systems with piecewise linear gain functions which are a simplification of the nonlinear bell-shaped gain curves of the original systems. For PFL, the gain curve is an isosceles triangle with a height of $G_{1\max}$ and base length of $2W_1$, while the inverted bell-shaped gain curve for NFL is an isosceles triangle with a height of $|G_{\min}|$ and base length of $2W_2$. Given the PFL should have a bifurcation diagram of type II, $G_{1\max} > 1$. Next, we derive the conditions for different types of bifurcation diagrams with those piecewise linear gain curves.

6.1 Case 1: $G_{1\max} + G_{2\min} > 1$

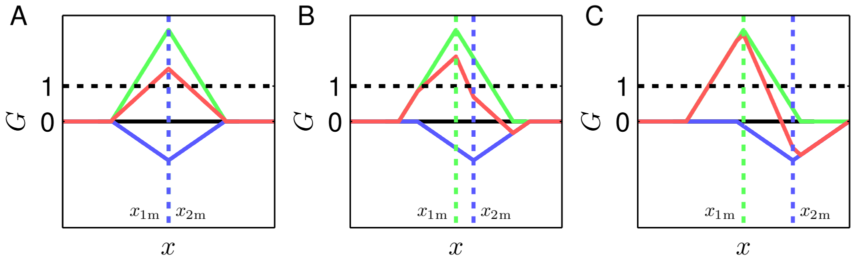

FIG. 4: Illustration for the simplified $G_1(x)$, $G_2(x)$ and $G_{\mathrm{C}}(x)$ curves with $G_{1\max} + G_{2\min} > 1$ and $0 < \frac{W_1}{W_2} < r_1 \equiv -\frac{G_{1\max}}{G_{2\min}}$. Only $x_{2\mathrm{m}}$ is varied for different panels.

If $0 < \frac{W_1}{W_2} < r_1 \equiv -\frac{G_{1\max}}{G_{2\min}}$, the G_{C}(x) curve has only two intersections with $G(x) = 1$ for any R (Fig. 4). Thus, the coupled system always admits the bifurcation diagram of type II.

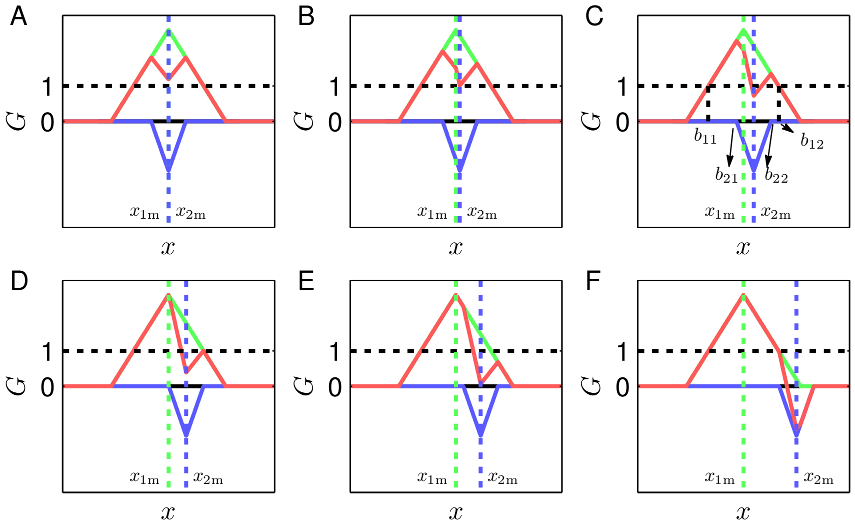

FIG. 5: Illustration for the simplified $G_1(x)$, $G_2(x)$ and $G_{\mathrm{C}}(x)$ curves with $G_{1\max} + G_{2\min} > 1$ and $\frac{W_1}{W_2} > r_1 \equiv -\frac{G_{1\max}}{G_{2\min}}$. Only $x_{2\mathrm{m}}$ is varied for different panels.

If $\frac{W_1}{W_2} > r_1$, the G_{C} curve intersects with $G(x) = 1$ four times within a limited range of R (Fig. 5C). The lower threshold of $|R|$ for four intersections, $R_3^{'}$, equals $\frac{G_{1\max}+G_{2\min}-1}{G_{1\max}}W_1$ when the middle minimum of G_C just equals 1.0 (Fig. 5B), while the upper threshold, $R_4^{'}$, is $\frac{G_{1\max}-1}{G_{1\max}}W_1 - W_2$ when one of the local maxima of G_C equals 1.0 (Fig.

5D). For $|R| < R_3^{'}$ or $|R| > R_4^{'}$, only two intersections are allowed and the coupled system can admit bistability rather than tristability.

To determine whether the bifurcation diagram is of type III or IV–VII when $R_3^{'} < |R| < R_4^{'}$, we calculate $\ln(\eta_4/\eta_1)$, where η_1 and η_4 are the values of η at SN_1 and SN_4. If $\ln(\eta_4/\eta_1) < 0$, two bistability regions overlap and tristability appears (Fig. 7D in the main text). If $\ln(\eta_4/\eta_1) > 0$, two bistability regions are separate and no tristability is allowed (Fig. 7F in the main text).

Notably,

$$\begin{aligned} \ln(\eta_4/\eta_1) &= \ln \frac{b_{12}}{b_{11}} \frac{\tilde{T_\mathrm{C}}(b_{11})}{\tilde{T_\mathrm{C}}(b_{12})} \\ &= [\ln \tilde{T_\mathrm{C}}(b_{11}) - \ln \tilde{T_\mathrm{C}}(b_{12})] - (\ln b_{11} - \ln b_{12}). \end{aligned} \tag{81}$$

With $G_\mathrm{C} = \frac{d \ln \tilde{T_\mathrm{C}}(x)}{d \ln x}$,

$$\begin{aligned} \ln(\eta_4/\eta_1) &= \int_{b_{12}}^{b_{11}} G_\mathrm{C}(x) d\ln x - (\ln b_{11} - \ln b_{12}) \\ &= \int_{b_{12}}^{b_{11}} G_1(x) d\ln x - (\ln b_{11} - \ln b_{12}) + \int_{b_{22}}^{b_{21}} G_2(x) d\ln x \\ &= -W_1(G_{1\mathrm{max}} + G_{1\mathrm{max}}^{-1} - 2) - W_2 G_{2\mathrm{min}}. \end{aligned} \tag{82}$$

Thus, if $\ln(\eta_4/\eta_1) < 0$, i.e., $\frac{W_1}{W_2} > r_3 \equiv -\frac{G_{2\mathrm{min}}}{G_{1\mathrm{max}} + G_{1\mathrm{max}}^{-1} - 2}$, the bifurcation diagrams of type IV–VII can be produced and tristability is allowed; if $\ln(\eta_4/\eta_1) > 0$, i.e., $0 < \frac{W_1}{W_2} < r_3$, only type III is possible and tristability cannot appear. However, $r_1 > r_3$ always holds true for $G_{1\mathrm{max}} + G_{2\mathrm{min}} > 1$; if $\frac{W_1}{W_2} > r_1$, tristability can appear via the bifurcation diagrams of type IV–VII when $R_3^{'} < |R| < R_4^{'}$.

6.2 Case 2: $G_{1\mathrm{max}} + G_{2\mathrm{min}} < 1$

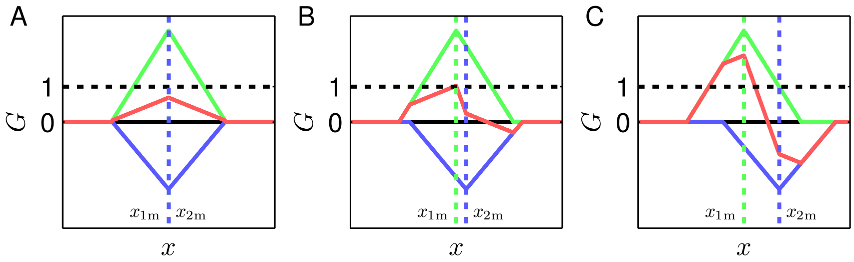

FIG. 6: Illustration for the simplified $G_1(x)$, $G_2(x)$ and $G_\mathrm{C}(x)$ curves with $G_{1\mathrm{max}} + G_{2\mathrm{min}} < 1$ and $0 < \frac{W_1}{W_2} < r_1 \equiv \frac{G_{1\mathrm{max}}}{G_{1\mathrm{max}} - 1}$. Only $x_{2\mathrm{m}}$ is varied for different panels.

If $0 < \frac{W_1}{W_2} < r_1 = -\frac{G_{1\mathrm{max}}}{G_{2\mathrm{min}}}$, the G_C curve can have at most two intersections with $G(x) = 1$, and thus bistability is allowed but tristability is forbidden (Fig. 6). When $|R|$ approaches 0, the maximum of G_C becomes less than 1 due to $G_{1\mathrm{max}} + G_{2\mathrm{min}} < 1$, and bistability also disappears (Fig. 6A). The threshold of R for bistability is $R_5^{'} \equiv \frac{G_{1\mathrm{max}} + G_{2\mathrm{min}} - 1}{G_{2\mathrm{min}}} W_2$ when the maximum of G_C equals 1 (Fig. 6B). If $|R| < R_5^{'}$, only the bifurcation diagram of type I is allowable; if $|R| > R_5^{'}$, type II is admitted and bistability is possible (Fig. 6C).

If $r_1 < \frac{W_1}{W_2} < r_2 \equiv \frac{G_{1\mathrm{max}}}{G_{1\mathrm{max}} - 1}$, two local maxima in the G_C curve are less than 1.0 for $R = 0$ (Fig. 7A). When R is non-zero, one maximum rises but the other drops. Thus, the G_C curve still has at most two intersections with $G(x) = 1$. However, the threshold of R for bistability is $R_6^{'} \equiv W_2 - \frac{G_{1\mathrm{max}} - 1}{G_{1\mathrm{max}}} W_1$ when the larger of the two maxima of G_C equals 1 (Fig. 7B). If $|R| < R_6^{'}$, only the bifurcation diagrams of type I can be produced; if $|R| > R_6^{'}$, type II is admitted and bistability is allowable (Fig. 7C).

If $\frac{W_1}{W_2} > r_2$, the G_C curve can have four intersections with $G(x) = 1$ when $|R|$ approaches 0, indicating the possibility of tristability (Fig. 8A). However, when $|R|$ is much larger, there are only two intersections, and thus only bistability

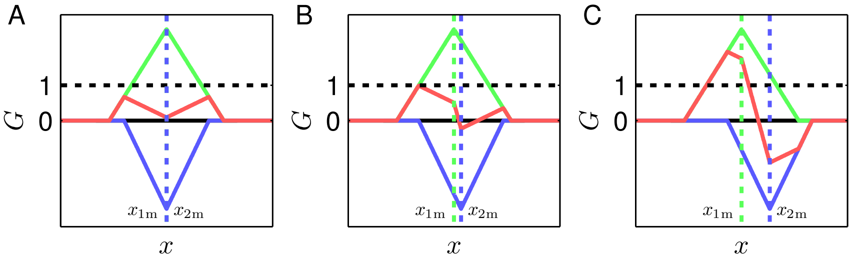

FIG. 7: Illustration for the simplified $G_1(x)$, $G_2(x)$ and $G_{\mathrm{C}}(x)$ curves with $G_{1\max} + G_{2\min} < 1$ and $r_1 < \frac{W_1}{W_2} < r_2 \equiv \frac{G_{1\max}}{G_{1\max}-1}$. Only $x_{2\mathrm{m}}$ is varied for different panels.

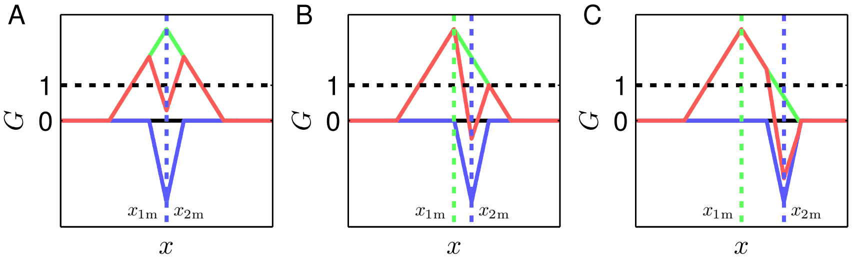

FIG. 8: Illustration for the simplified $G_1(x)$, $G_2(x)$ and $G_{\mathrm{C}}(x)$ curves with $G_{1\max} + G_{2\min} < 1$ and $\frac{W_1}{W_2} > r_2$. Only $x_{2\mathrm{m}}$ is varied for different panels.

arises (Fig. 8C). The threshold of R, used to discriminate between these two cases, is $R_7' \equiv \frac{G_{1\max}-1}{G_{1\max}} W_1 - W_2$ when the smaller maximum of G_{C} equals 1 (Fig. 8B). If $|R| > R_7'$, only the bifurcation diagrams of type II are allowed; otherwise, either type III or type IV–VII is admitted.

It is noted that $r_3 > r_2$ always holds for $G_{1\max} + G_{2\min} < 1$. As mentioned above, the relationship between $\frac{W_1}{W_2}$ and r_3 determines whether the bifurcation diagram is of type III or IV–VII. Thus, if $\frac{W_1}{W_2} > r_3$, type IV–VII is allowed and tristability is possible; if $r_2 < \frac{W_1}{W_2} < r_3$, only type III is admitted.

7. ANALYSIS OF TWO OTHER TYPES OF MULTIPLICATIVELY COUPLED FEEDBACK LOOPS

Although the results in the main text are based on a particular type of coupled feedback loops, the analysis method is also applicable to at least two types of coupled feedback loops given the loops are multiplicatively coupled.

A mutual inhibitory loop combined with a self-regulation loop

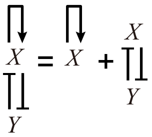

FIG. 9: Schematic of a mutual inhibitory loop combined with a self-regulation loop. The system can be decomposed into two separate loops. The arrow- and bar-headed lines denote activation and inhibition, respectively.

Such a motif often contains two components denoted by X and Y. Their concentrations are x and y, respectively.

The kinetic equations for this motif are as follows:

$$\frac{dx}{d\tau} = \eta f(x)g(y) - x, \quad (83)$$

$$\theta_y \frac{dy}{d\tau} = h(x) - y, \quad (84)$$

where $f(x)$ describes the autoregulation of X, while $g(y)$ and $h(x)$ characterize the mutual regulation between X and Y.

For two individual loops in the system, the transfer functions are

$$T_1(x) = \eta f(x), \quad (85)$$

and

$$T_2(x) = (\eta g \circ h)(x). \quad (86)$$

Thus, the corresponding logarithmic gains are

$$G_1(x) = \frac{x}{f(x)} \frac{d}{dx}[f(x)], \quad (87)$$

and

$$G_2(x) = \frac{x}{g \circ h(x)} \frac{d}{dx}[g \circ h(x)], \quad (88)$$

respectively. For the coupled feedback loops, the transfer function and gain are separately

$$T_C(x) = [\eta f(g \circ h)](x), \quad (89)$$

and

$$\begin{aligned} G_C(x) &= \frac{x}{f(x)} \frac{d}{dx} f(x) + \frac{x}{g \circ h(x)} \frac{d}{dx}[g \circ h(x)] \\ &= G_1(x) + G_2(x). \end{aligned} \quad (90)$$

Denote the steady-state value by x. The characteristic equation for the coupled loops is

$$\lambda^2 + A_{\mathrm{C}}\lambda + B_{\mathrm{C}} = 0, \quad (91)$$

with

$$\begin{aligned} A_{\mathrm{C}} &= 1 + \frac{1}{\theta_y} - \frac{x}{f(x)} \frac{d}{dx} f(x), \\ B_{\mathrm{C}} &= \frac{1}{\theta_y}\{1 - \frac{x}{f(x)} \frac{d}{dx} f(x) - \frac{x}{g \circ h(x)} \frac{d}{dx}[g \circ h(x)]\} \\ &= \frac{1}{\theta_y}[1 - G_1(x) - G_2(x)]. \end{aligned}$$

By setting $\lambda = 0$, we obtain the necessary condition for SN bifurcation:

$$G_1(x) + G_2(x) = 1. \quad (92)$$

Two mutual inhibitory loops sharing one component

This type of motif usually contains three components. Denote them by X, Y and Z, and their concentrations by x, y and z, respectively. The kinetic equations for this motif are as follows:

$$\frac{dx}{d\tau} = \eta f(y)g(z) - x, \quad (93)$$

$$\theta_y \frac{dy}{d\tau} = h(x) - y, \quad (94)$$

$$\theta_z \frac{dz}{d\tau} = u(x) - y, \quad (95)$$

(96)

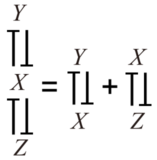

FIG. 10: Schematic of two mutual inhibitory loops sharing one component. The system can be decomposed into two loops. The arrow- and bar-headed lines denote activation and inhibition, respectively.

where $f(y)$ and $h(x)$ describe the mutual regulation between X and Y, while $g(z)$ and $u(x)$ characterize that between X and Z.

For two individual loops in the system, the transfer functions are

$$T_1(x) = [\eta f \circ h](x), \tag{97}$$

and

$$T_2(x) = (\eta g \circ u)(x). \tag{98}$$

Thus, the corresponding logarithmic gains are

$$G_1(x) \;=\; \frac{x}{f \circ h(x)} \frac{d}{dx}[f \circ h(x)], \tag{99}$$

and

$$G_2(x) \;=\; \frac{x}{g \circ u(x)} \frac{d}{dx}[g \circ u(x)], \tag{100}$$

respectively. For the coupled loops, the transfer function and gain are separately

$$T_C(x) \;=\; [\eta (f \circ h)(g \circ u)](x), \tag{101}$$

and

$$\begin{aligned} G_C(x) &= \frac{x}{f \circ h(x)} \frac{d}{dx}[f \circ h(x)] + \frac{x}{g \circ u(x)} \frac{d}{dx}[g \circ u(x)] \\ &= G_1(x) + G_2(x). \end{aligned} \tag{102}$$

Denote by x the steady-state value. The characteristic equation for the coupled feedback loops is

$$\lambda^3 + A_{\rm C}\lambda^2 + B_{\rm C}\lambda + C_{\rm C} \;=\; 0, \tag{103}$$

where

$$\begin{aligned} A_{\rm C} &= \frac{1}{\theta_y} + \frac{1}{\theta_z} + 1, \\ B_{\rm C} &= \frac{1}{\theta_y}\{1 - \frac{x}{f \circ h(x)} \frac{d}{dx}[f \circ h(x)]\} + \frac{1}{\theta_z}\{1 - \frac{x}{g \circ u(x)} \frac{d}{dx}[g \circ u(x)]\} + \frac{1}{\theta_y \theta_z}, \\ C_{\rm C} &= \frac{1}{\theta_y \theta_z}\{1 - \frac{x}{f \circ h(x)} \frac{d}{dx}[f \circ h(x)] - \frac{x}{g \circ u(x)} \frac{d}{dx}[g \circ u(x)]\} \\ &= \frac{1}{\theta_y \theta_z}[1 - G_1(x) - G_2(x)]. \end{aligned}$$

By setting $\lambda = 0$, we obtain the necessary condition for SN bifurcation:

$$G_1(x) + G_2(x) = 1. \tag{104}$$

[1] T. S. Gardner, C. R. Cantor and J. J. Collins. *Construction of a genetic toggle switch in escherichia coli.* Nature **403**, 339 (2000).

[2] H. Ge, H. Qian and X. S. Xie. *Stochastic phenotype transition of a single cell in an intermediate region of gene state switching.* Phys. Rev. Lett. **114**, 078101 (2015).
[3] V. Shahrezaei and P. S. Swain. *Analytical distributions for stochastic gene expression.* Proc. Natl. Acad. Sci. U. S. A. **105**, 17256 (2008).
[4] S. Bail, M. Swerdel, H. Liu, X. Jiao, L. A. Goff, R. P. Hart and M. Kiledjian. *Differential regulation of microrna stability.* RNA **16**, 1032 (2010).
[5] A. Tiwari and O. A. Igoshin. *Coupling between feedback loops in autoregulatory networks affects bistability range, open-loop gain and switching times.* Phys. Biol. **9**, 055003 (2012).
[6] J. C. Ray and O. A. Igoshin. *Adaptable functionality of transcriptional feedback in bacterial two-component systems.* PLoS Comput. Biol. **6**, e1000676 (2010).
[7] A. Tiwari, G. Balázsi, M. L. Gennaro and O. A. Igoshin. *The interplay of multiple feedback loops with post-translational kinetics results in bistability of mycobacterial stress response.* Phys. Biol. **7**, 036005 (2010).
[8] B. Ananthasubramaniam and H. Herzel. *Positive feedback promotes oscillations in negative feedback loops.* PLoS One **9**, e104761 (2014).
[9] B. Novak and J. Tyson. *Design principles of biochemical oscillators.* Nat. Rev. Mol. Cell Biol. **9**, 981-991 (2008).
[10] B. Pfeuty and K. Kaneko. *The combination of positive and negative feedback loops confers exquisite flexibility to biochemical switches.* Phys. Biol. **6**, 46013 (2009).

II. SUPPLEMENTAL FIGURES

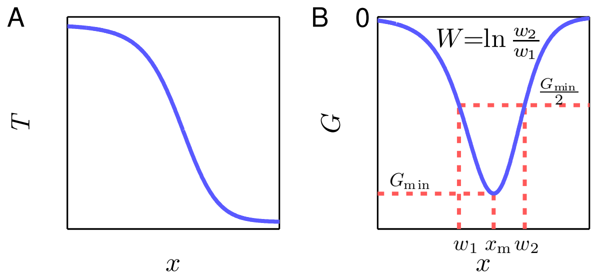

FIG. S1: Characteristics of individual NFLs in terms of gain. (A) Transfer function T is characterized by a reverse sigmoid function. (B) Logarithmic gain G. x-axis and T-axis are in log scale.

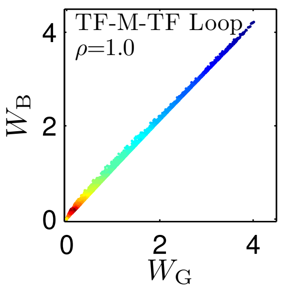

FIG. S2: Scatter diagram of W_{B} versus $W_{\mathrm{G}} \equiv W(G_{\max} + \frac{1}{G_{\max}} - 2)$. Each dot represents one TF-M-TF loop. From blue to red, more dots are distributed locally. The Pearson's correlation coefficient is 1.0.

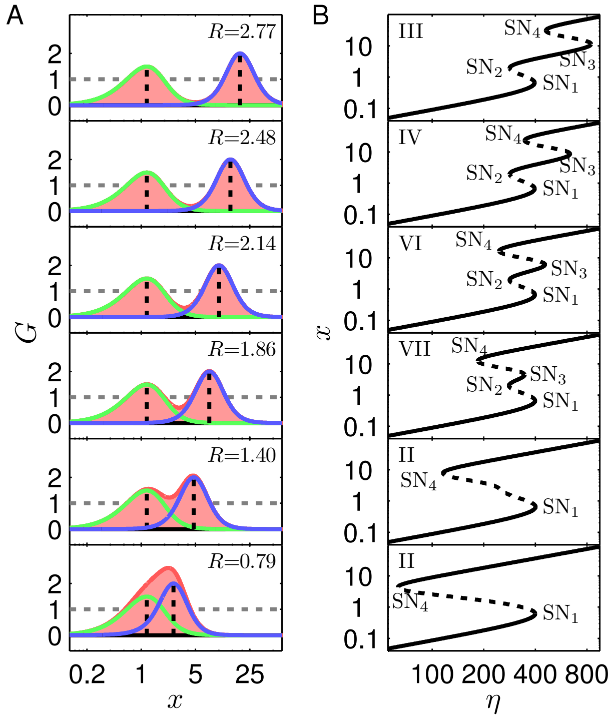

FIG. S3: Effects of R on G_C and bifurcation diagrams for System 2. (A) The curves of G_1 (green), G_2 (blue) and G_C (red); (B) the corresponding bifurcation diagrams, where the solid and dashed curves denote stable and unstable steady states, respectively. The four bifurcation points and the corresponding η values are marked. The x-axis and η-axis are in log scale.

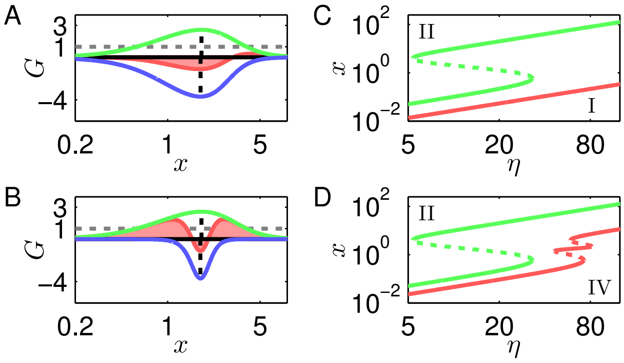

FIG. S4: Effects of full width at half extremum on G_C and bifurcation diagram of $P_{II}N$. (A–B) The curves of G_1 (green), G_2 (blue) and G_C (red) for different values of W_2; (C–D) the corresponding bifurcation diagrams of the TF-TF PFL (green) and $P_{II}N$ (red). (A, C) and (B, D) correspond to Systems 3 and 4, respectively. The x-axis and η-axis are in log scale.

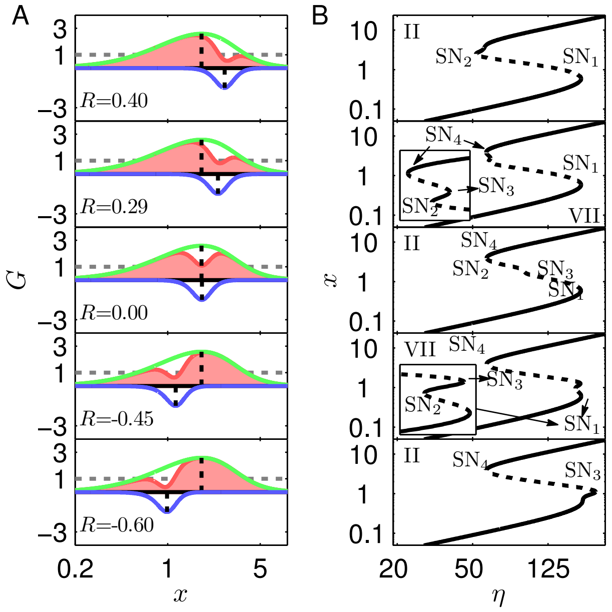

FIG. S5: Effects of R on G_C and bifurcation diagrams of $P_{II}N$ (System 5). (A) The curves of G_1 (green), G_2 (blue) and G_C (red); (B) the corresponding bifurcation diagrams, where the solid and dashed curves denote stable and unstable steady states, respectively. The four bifurcation points and the corresponding η values are marked. The x-axis and η-axis are in log scale.

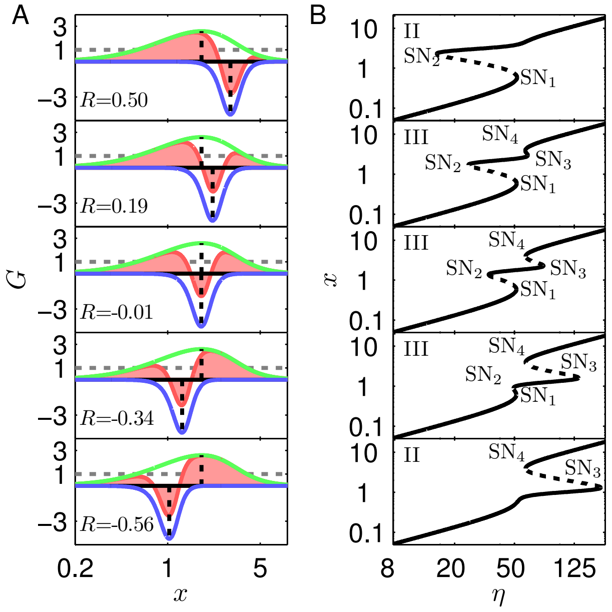

FIG. S6: Effects of R on G_C and bifurcation diagrams of $P_{II}N$ (System 6). (A) The curves of G_1 (green), G_2 (blue) and G_C (red); (B) the corresponding bifurcation diagrams, where the solid and dashed curves denote stable and unstable steady states, respectively. The four bifurcation points and the corresponding η values are marked. The x-axis and η-axis are in log scale.

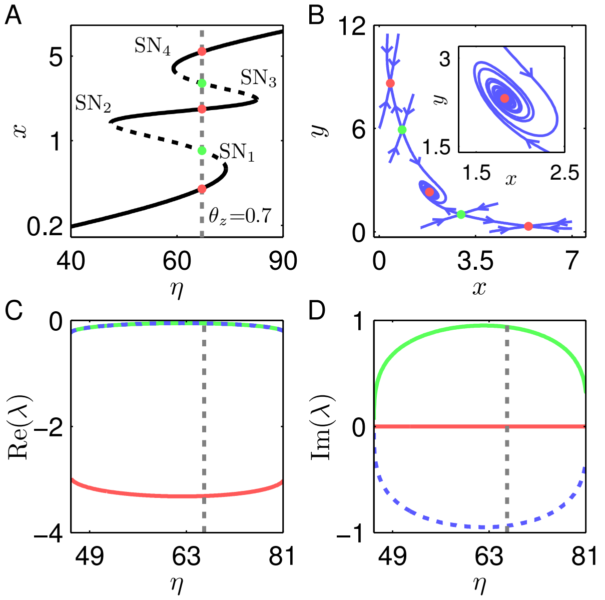

FIG. S7: Dynamics of $P_{II}N$ (System 4 with $R = -0.02$). (A) Bifurcation diagram. The black solid and dashed curves denote stable and unstable steady states, respectively. (B) The phase portraits at $\eta = 66$. The blue curves with arrows denote orbits, and the red and green dots separately denote stable and unstable steady states. The inset is a zoom in of the orbits around the intermediate steady state. (C, D) Three eigenvalues at the stable steady states between SN_2 and SN_3 in (A). The vertical dashed line denotes $\eta = 66$. The x-axis and η-axis are in log scale.

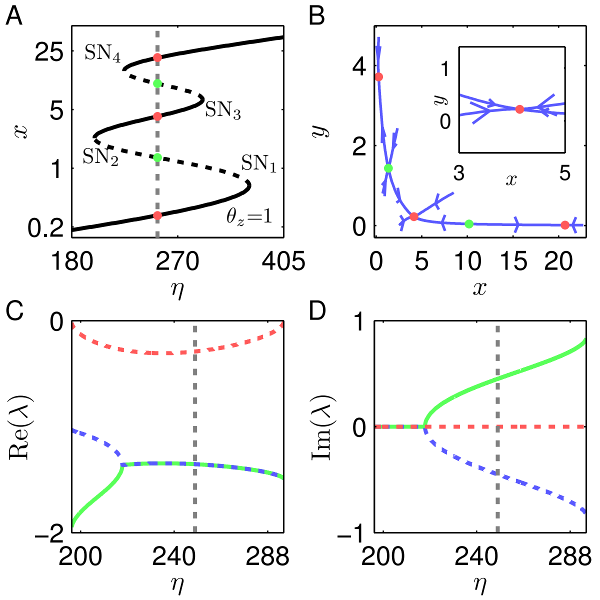

FIG. S8: Dynamics of $P_{II}P_{II}$ (System 1 with $R = 2.04$). (A) Bifurcation diagram. The black solid and dashed curves denote stable and unstable steady states, respectively. (B) The phase portraits at $\eta = 250$. The blue curves with arrows denote orbits, and the red and green dots separately denote stable and unstable steady states. The inset is a zoom in of the orbits around the intermediate steady state. (C, D) Three eigenvalues at the stable steady states between SN_2 and SN_3 in (A). The vertical dashed line denotes $\eta = 250$. The x-axis and η-axis are in log scale.

III Supplementary Tables

Table S1 The sampling ranges of the parameters

	n_1	n_2	n_3	δ	K	α	κ
PP	1–10	1–10	1–10	0.1–100	0.01–100	1–100	0.1–100
PN	1–10	1–10	1–10	0.1–100	0.01–100	0.01–1	0.1–100

Parameter sets are sampled uniformly on a logarithmic scale in a 7-dimensional parameter space using the Latin hypercube sampling method. Parameters θ_z and θ_y are kept at 1.0.

Table S2 The percentage of each type of bifurcation diagram for different kinds of coupled feedback loops

	P_IP_I	P_IP_{II} & $P_{II}P_I$	$P_{II}P_{II}$	P_IN	$P_{II}N$
I	97.2	0	0	100	1.6
II	2.8	99.6	61.5	0	95.9
III	<0.01	0.1	1.5	0	0.3
IV–VII	<0.01	0.3	37.0	0	2.2

P_I and P_{II} denote the individual PFLs with bifurcation diagrams of type I and II, respectively. P_IP_I, P_IP_{II}, $P_{II}P_I$ and $P_{II}P_{II}$ denote different kinds of dual-PFL systems, while P_IN and $P_{II}N$ denote two kinds of coupled PFL and NFL systems. The first and second items denote the types of TF-TF loop and TF-M-TF loop, respectively.

System	Features	x_{2m}	R	n_3*	K*	α*	κ	θ_z
1	$P_{II}P_{II}$<br>G_{1max} 1.73<br>x_{1m} 1.28<br>W_1 1.46<br>G_{2max} 1.60<br>W_2 0.96	17.56	2.62	1.98	0.020	6.02	4	1
		13.94	2.39	1.99	0.032	6.02	4	1
		9.85	2.04	2.00	0.063	6.03	4	1
		7.81	1.81	2.01	0.10	6.05	4	1
		4.34	1.22	2.06	0.32	6.13	4	1
		2.66	0.73	2.21	0.79	6.35	4	1
2	$P_{II}P_{II}$<br>G_{1max} 1.49<br>x_{1m} 1.18<br>W_1 1.44<br>G_{2max} 2.00<br>W_2 0.99	18.78	2.77	1.99	0.016	9.96	3.2	1
		14.14	2.48	2.00	0.028	9.97	3.2	1
		10.05	2.14	2.01	0.055	9.99	3.2	1
		7.56	1.86	2.02	0.097	10.13	3.2	1
		4.76	1.40	2.06	0.24	10.17	3.2	1
		2.61	0.79	2.23	0.74	10.76	3.2	1
3	$P_{II}N$<br>G_{1max} 2.59<br>x_{1m} 1.81<br>W_1 1.62<br>G_{2min} -3.70<br>W_2 1.37	1.77	-0.02	2.80	1.19	1.19e-4	10	0.7
				δ=1000				
4	$P_{II}N$<br>G_{1max} 2.59<br>x_{1m} 1.81<br>W_1 1.62<br>G_{2min} -3.70<br>W_2 0.39	2.57	0.35	5.58	1.15	0.120	10	0.7
		2.00	0.10	6.05	1.78	0.119	10	0.7
		1.77	-0.02	6.38	2.17	0.119	10	0.7
		1.35	-0.29	7.47	3.24	0.117	10	0.7
		0.98	-0.61	9.90	4.84	0.114	10	0.7
5	$P_{II}N$<br>G_{1max} 2.59<br>x_{1m} 1.81<br>W_1 1.62<br>G_{2min} -1.50<br>W_2 0.39	2.69	0.40	5.18	1.15	0.465	10	0.7
		2.40	0.29	5.34	1.41	0.465	10	0.7
		1.81	0.00	5.92	2.24	0.463	10	0.7
		1.15	-0.45	7.95	4.23	0.460	10	0.7
		0.99	-0.60	9.15	5.00	0.458	10	0.7
6	$P_{II}N$<br>G_{1max} 2.59<br>x_{1m} 1.81<br>W_1 1.62<br>G_{2min} -4.50<br>W_2 0.39	2.98	0.50	5.55	0.86	0.0573	10	0.7
		2.19	0.19	6.02	1.49	0.0565	10	0.7
		1.79	-0.01	6.52	2.08	0.0556	10	0.7
		1.29	-0.34	7.98	3.42	0.0536	10	0.7
		1.03	-0.56	9.67	4.50	0.0517	10	0.7

*For each system, the values of n_3, α and K are changed such that only x_{2m} is altered while the other characteristic quantities, i.e., G_{1max}, x_{1m}, W_1, G_{2max} (G_{2min}), and W_2 are fixed. The parameters besides those shown in the table are always set as follows: n_1=2, n_2=2, δ=10, and θ_y=1.
